# Comparative Nitrene-Transfer Chemistry to Olefins Mediated by First-Row Transition Metal Catalysts Supported by a Pyridinophane Macrocycle with N4 Ligation

**DOI:** 10.3390/molecules30153097

**Published:** 2025-07-24

**Authors:** Himanshu Bhatia, Lillian P. Adams, Ingrid Cordsiemon, Suraj Kumar Sahoo, Amitava Choudhury, Thomas R. Cundari, Pericles Stavropoulos

**Affiliations:** 1 Department of Chemistry, Missouri University of Science and Technology, Rolla, MO 65409, USA; hbgxg@umsystem.edu (H.B.); lillian.adams.1@slu.edu (L.P.A.); iccord3@gmail.com (I.C.); surajsahoo2392@gmail.com (S.K.S.); choudhurya@mst.edu (A.C.); 2 Department of Chemistry, Center for Advanced Scientific Computing and Modelling (CASCaM), University of North Texas, Denton, TX 76203, USA

**Keywords:** nitrene transfer, pyridine-containing macrocycles, aziridination, olefins, copper nitrenes, electrophilicity

## Abstract

A 12-membered pyridinophane scaffold containing two pyridine and two tertiary amine residues is examined as a prototype ligand (^tBu^N4) for supporting nitrene transfer to olefins. The known [(^tBu^N4)M^II^(MeCN)_2_]^2+^ (M = Mn, Fe, Co, and Ni) and [(^tBu^N4)Cu^I^(MeCN)]^+^ cations are synthesized with the hexafluorophosphate counteranion. The aziridination of para-substituted styrenes with PhI=NTs (Ts = tosyl) in various solvents proved to be high yielding for the Cu(I) and Cu(II) reagents, in contrast to the modest efficacy of all other metals. For α-substituted styrenes, aziridination is accompanied by products of aziridine ring opening, especially in chlorinated solvents. Bulkier β-substituted styrenes reduce product yields, largely for the Cu(II) reagent. Aromatic olefins are more reactive than aliphatic congeners by a significant margin. Mechanistic studies (Hammett plots, KIE, and stereochemical scrambling) suggest that both copper reagents operate via sequential formation of two N–C bonds during the aziridination of styrene, but with differential mechanistic parameters, pointing towards two distinct catalytic manifolds. Computational studies indicate that the putative copper nitrenes derived from Cu(I) and Cu(II) are each associated with closely spaced dual spin states, featuring high spin densities on the nitrene N atom. The computed electrophilicity of the Cu(I)-derived nitrene reflects the faster operation of the Cu(I) manifold.

## 1. Introduction

Aza-macrocycles present advantages over acyclic ligand frameworks in atom/group transfer chemistry (e.g., oxo, nitrene, and carbene), associated, inter alia, with thermodynamic stability, functional group rigidity, enhanced metal-binding capability, and the potential for orthogonal orientation of the putative metal-atom/group moiety versus the macrocyclic platform [1,2,3]. It is not coincidental that biological systems make extensive use of porphyrins for supporting, for instance, powerful iron-oxo units in selective C–H bond insertion chemistry [4], setting a paradigm for developing biomimetic systems with the assistance of various porphyrin scaffolds and related *π*-conjugated macrocycles (e.g., porphyrazines, corroles, and corrolazines) [1,5,6,7,8,9,10,11]. Genetically engineered heme proteins have also been advanced for the catalytic transfer of groups (nitrenes and carbenes) that are rarely encountered in physiological settings [12,13,14,15].

**Scheme 1:**
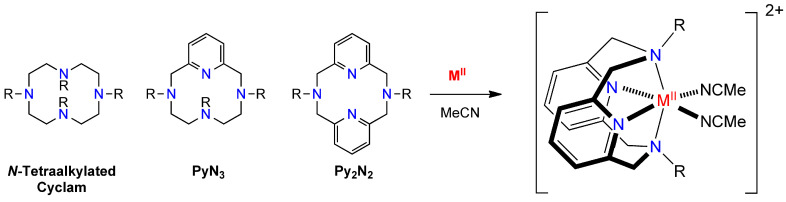
Introduction of pyridine into 12-membered *N*-tetraalkylated cyclams.

Non-heme macrocyclic platforms have more recently been developed for applications in selective atom/group-transfer biomimetic chemistry [16]. Inspirational advances have been achieved with tetraamido macrocyclic ligands (TAML) [17,18,19,20,21] and tetra-N-heterocyclic carbene macrocycles in oxo- and nitrene-transfer chemistry [22,23]. Another category of polyazamacrocycles of great interest to the present work (Scheme 1) originates from *N*-tetraalkylated cyclams of various ring sizes (commonly *n* = 12–14) [24], which have been further derivatized by replacing one (PyN_3_) or two (Py_2_N_2_) tertiary amine residues with pyridine (pyridinophanes) [25,26]. Among other applications, this class of tetraazamacrocycles has found extensive use in stoichiometric and catalytic metal/O_2_ (or H_2_O_2_) chemistry [27,28,29,30,31] with first-row transition elements. One member of the pyridinophane group, combining two pyridine and two tertiary amine residues (Py_2_N_2_) in its 12-membered ring [32], has been rigorously investigated as a metalated scaffold (Mn, Co, and Ni) for the generation of metal-peroxo or -hydroxo species [33] with applications in the hydroxylation of aliphatics/aromatics [34,35,36], catalase or water oxidation activity [37,38,39], oxidation of aldehydes to carboxylic acid [40], and nitrile activation [41,42,43]. Upon metalation, this compact 12-membered pyridinophane macrocycle undergoes significant folding, placing the pyridine moieties in equatorial positions and the tertiary amine residues usually in elongated axial sites (Scheme 1) [44]. For divalent metals, two additional equatorial sites can be readily occupied by two cis-oriented solvent molecules, hence giving rise to the potential of conducting cross-coupling chemistry, as evidenced in studies of Ni- or Pd-mediated C–C and C–O oxidative coupling, as well as C–C bond cleavage, in the presence of O_2_ or H_2_O_2_ [45,46,47,48,49]. Moreover, instances of $C_{{\mathrm{sp}}^{3}}$–H bond activation have been reported by means of Pd(II) species [50]. Notables are also reductive approaches, especially those with regards to the electro- or photo-catalytic oxygen reduction reaction (ORR) mediated by Pd(III) or Co(III) intermediates [51,52]. Finally, several pyridinophanecopper(I) complexes exhibit valuable photoluminescent properties [53,54].

In the present publication, we examine the Py_2_N_2_ scaffold as a supporting ligand for comparative nitrene transfer to olefins, mediated by first-row transition elements (Mn^II^, Fe^II^, Co^II^, Ni^II^, and Cu^I/II^). An analogous systematic study of Cu(I)-catalyzed carbene addition/insertion to olefins and X–H bonds (X = Si, O, and N) is available with 12-membered pyridinophanes featuring only one pyridine residue (PyN_3_) [25,26,55,56,57]. Our ultimate goal consists of capitalizing on the modular synthesis of the Py_2_N_2_ framework [58], enabling facile insertion of pyridine substituents at the para position (electronic effect) and various alkyl groups as tert-amine substituents (steric effect), to support stereoselective (including enantioselective) metal-dependent nitrene-transfer chemistry. As an initial point of departure, we select to explore a commonly employed member of the Py_2_N_2_ ligand family, featuring t-Bu substitution of the tert-amine residues (usually abbreviated as ^tBu^N4) [44]. As a ligand, ^tBu^N4 is known to be redox innocent from +1.7 V to approximately −2.0 V (Zn^II^ complex; vs. Fc^+^/Fc) and capable of stabilizing both low (e.g., Co^I^ and Ni^I^) and high (e.g., Mn^IV^, Ni^III/IV^, and Pd^III/IV^) oxidation states in various complexes [44,47,59,60,61]. In the present study, we aspire to (i) determine the nitrene-transfer capabilities of several ^tBu^N4-supported first-row transition metal sites with respect to olefins (X–H insertions will be addressed separately); and (ii) provide an initial appreciation of the mode of operation of putative metal-nitrene moieties associated with the most successful catalysts.

## 2. Results and Discussion

**Scheme 2:**
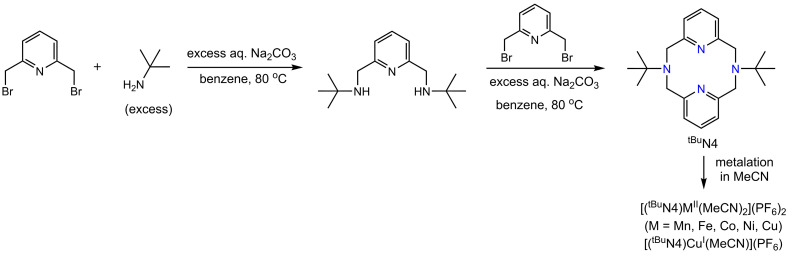
Synthesis of ligand and metal compounds used in this study.

**Synthesis of Ligands and Compounds.** The known ligand ^tBu^N4 was prepared according to a reported literature procedure [62], with a small modification in the first step, involving use of excess t-butyl amine (30 equiv.) over 2,6-bis(bromomethyl)pyridine to avoid the formation of higher-order macrocycles (Scheme 2).

The cationic components of all compounds employed as catalysts in this study, namely [(^tBu^N4)M^II^(MeCN)_2_]^2+^ (M = Mn, Fe, Co, and Ni) and [(^tBu^N4)Cu^I^(MeCN)]^+^, are known entities with various counteranions [44]. Given the extensive use of PF_6_^−^ in previous studies from our group, we synthesized all compounds noted above as hexafluorophosphates. Those for Cu^I^ and Mn^II^ are previously known [38,54], but all others are new compounds that have been synthesized from the reaction of the corresponding anhydrous MCl_2_ with the ligand (^tBu^N4) in acetonitrile, followed by chloride abstraction (single pot) with the assistance of TlPF_6_. Specifically for the nickel compound, the intermediate [(^tBu^N4)Ni^II^Cl_2_] was first isolated. All new compounds were characterized using various means (UV–vis, IR, and CHN elemental analysis), including single-crystal X-ray diffraction analysis of crystals obtained by layering diethyl ether over acetonitrile solutions of the complexes at −35 °C.

Unsurprisingly, structural characterization of all new compounds (Crystallographic Data in Table S1; ORTEPs in Figure S1) reveals similar features to those detailed for the analogous series of complexes with triflate (TfO^–^) as counteranion [44]. Table S2 summarizes key metrical parameters. The usual distorted octahedral coordination (axially elongated) with *^t^*BuN residues at the apex is observed for all divalent sites. As noted before, the most prominent feature is the tighter ligand field exerted in the case of Fe(II) and Co(II), with bond lengths even shorter than those observed in the triflate series, previously assigned to contributions from both high- and low-spin states [44].

**Catalytic Studies. (a) Aziridination of Styrene and Para-Substituted Styrenes.** To start evaluating the contribution of the ^tBu^N4-supported cationic metal complexes to nitrene-transfer chemistry, all divalent metal complexes [(^tBu^N4)M^II^(CH_3_CN)_2_](PF_6_)_2_ (M = Mn, Fe, Co, Ni, and Cu) as well as [(^tBu^N4)Cu^I^(CH_3_CN)](PF_6_) were first explored as catalysts for the aziridination of a panel of 4-X-styrenes (Table 1). As noted in a previous study from our lab [63], further observed in the present study, *dicationic* metal catalysts of base-metal elements can give rise to five-membered *N*-heterocycles (2,4-diaryl-N-tosylpyrrolidines) in addition to three-membered congeners (2-aryl-*N*-tosyl-aziridines), by virtue of an in situ (3 + 2) insertion of excess styrene into preformed aziridine. This additional reaction is more prominent in chlorinated solutions, by virtue of enhancing the acidity of the M(II) sites and enabling aziridine ring opening. To circumvent this complication, acetonitrile was selected as the solvent to evaluate the comparative efficacy and selectivity across the series of catalysts investigated in the present study. The catalytic reaction was accomplished by employing PhI=NTs (1.0 equiv.) as the nitrene source along with excess 4-X-styrene (8 equiv.) in the presence of the metal reagent (5 mol%) in acetonitrile as the solvent.

Under the same reaction conditions, all metal complexes mediate aziridination of the panel of para-substituted styrenes in variable yields (Table 1). Evidently, the Cu(I) and Cu(II) catalysts outperformed all other divalent metal reagents for the aziridination of all 4-X-styrenes examined. Overall, higher yields were observed with the Cu(I) catalyst compared to the Cu(II) catalyst, with only a few exceptions (X = Cl and F). In sharp contrast, all other divalent sites provide only modest yields, with a narrowly ranged reactivity trend of Fe ≥ Mn ≥ Ni ≈ Co. The slightly better efficacy of the dicationic Fe(II) species among base-metal elements in nitrene-transfer chemistry has also been noted in analogous studies with different N3- or N4-type ligand coordination [63,64,65,66,67].

The best-performing [(^tBu^N4)Cu^I^(CH_3_CN)](PF_6_) was further explored as a catalyst for the aziridination of para-substituted styrenes with PhINTs in both chlorinated (dichloromethane and 1,2-dichloroethane) and non-chlorinated (acetonitrile) solvents, in the presence of molecular sieves (5 Å) (Table 2). In all solvents examined, yields were very good (≥82%) and comparable for most substrates, irrespective of the electron-donating or -withdrawing character of the para-substituent. The two 4-alkoxy-substituted styrenes (MeO and *^t^*BuO) generated viscous solutions in chlorinated solvents, presumably featuring polymerized products, although good aziridine yields were obtained in acetonitrile.

**(b) Aziridination of other Aromatic and Aliphatic Olefins.** The most productive Cu(I) catalyst was further explored with a panel of aromatic olefins in dichloromethane, 1,2-dichloroethane, and acetonitrile under the conditions noted above (Table 3). The sterically and electronically hampered 2,4,6-trimethylstyrene (entry 1) affords more modest aziridination yields than the para-substituted styrenes, reflecting both steric and electronic encumbrance (orthogonal arene/alkene orientation) [68]. Steric effects are also observed to a lesser extent with α-substituted (Me, Ph) styrenes (entries 2, 3), featuring overall nitrene-transfer yields in the range of 52–72%. For α-Me-styrene (entry 2), the aziridination product dominates, especially in MeCN, accompanied by low yields of allylic amination, as well as enamine and hydroamination products. Conversely, the aziridination product of α-Ph-styrene (entry 3) is only a minor contributor, especially in chlorinated solvents. The corresponding enamine (major) and hydroamination (minor) products are observed instead, indicating that the origin of these products may be from a precursor benzylic carbocation generated upon initial nitrene addition to the β-carbon of the styrene (vide infra). Alternatively, aziridine ring opening (known for the nosyl aziridine) [69] may precede carbocation formation. For β-substituted (Me and Ph) styrenes (cis and trans, entries 4–7), overall yields observed are lower than those obtained for the α-substituted congeners, reflecting increased steric encumbrance. Whereas cis-β-Me-styrene (entry 4) affords both cis and trans aziridines (~1:1 ratio), the trans analog (entry 5) provides almost exclusively the corresponding trans aziridine (albeit in lower yields), reflecting the stability of trans aziridines. The retention of stereochemistry is even poorer for cis-stilbene (entry 6), although trans-stilbene (entry 7) affords trans aziridine exclusively, but in yields (≤20%) that further underscore steric encumbrance exerted by the phenyl group. A small amount of the enamine noted in the aziridination of 1,1-diphenylethylene (entry 3) is also observed in the case of cis-stilbene (entry 6) and assigned to aziridine ring opening and phenyl migration (known for the nosyl aziridine) [69]. Competition for aziridination versus allylic/benzylic amination (entries 8 and 9) favors the latter only if trans-olefins are involved and/or if conjugation is present (entry 8).

Compared to aromatic alkenes, aliphatic alkenes provide products of nitrene-transfer chemistry with significantly lower yields (entries 10–18). For instance, cycloalkenes (entries 10–12) afford aziridines and allylic amines in overall yields that exceed 20% only for the electron-rich cis-cyclooctene (entry 12). For this substrate, the corresponding aziridine is the sole product, as allylic amination is reportedly hampered due to poor *σ*_C–H_/*π*_C = C_ orbital overlap [70]. Similarly, methylenecyclohexane (entry 13) provides modest yields of aziridine (major) and allylic amine (single isomer). For substrates that feature one or more CH_2_ groups separating the alkene from the aromatic component (entries 14 and 15), aziridines are the major products, accompanied by allylic amines formed only for substrates that generate benzylic intermediates upon H-atom abstraction (entry 14). The mechanistically diagnostic cis-2-hexene affords cis and trans aziridines in <20% total yields and a cis/trans ratio of ~3 (entry 16). As usual, cis/trans selectivities tend to be superior for the aziridination of aliphatic alkenes, presumably due to facile oxidative aziridine-ring closure [71]. Similar yields are obtained for terminal alkenes (entries 17 and 18). Overall, the reactions performed in MeCN tend to generate lower amounts of byproducts resulting from aziridine ring opening, presumably due to the lower acidity of the catalytic metal sites in MeCN versus those in chlorinated solvents.

Selected substrates (entries 1–7, Table 3, yields in parentheses) were also tested in nitrene-transfer chemistry mediated by the Cu(II) catalyst [(^tBu^N4)Cu^II^(CH_3_CN)_2_](PF_6_)_2_ under the same reaction conditions applied for the Cu(I) congener in MeCN. Whereas the Cu(I) and Cu(II) precursors provided comparably high yields with para-substituted styrenes (Table 1 and Table 2), they diverged considerably as catalysts in the aziridination/amination of other bulkier olefins. Indeed, the absorption of PhI=NTs tends to be significantly slower with the Cu(II) precatalyst, and the corresponding yields are suppressed when significant steric encumbrance is present (ortho or β styrene substitution), even at prolonged reaction times. Indeed, low to modest yields are detected in the Cu(II)-mediated aziridination of 1,3,5-trimethylstyrene (entry 1), as well as for all β-substituted (Me and Ph) styrenes (entries 4–7). In particular, phenyl and trans substitution lead to very low yields, reflecting the steric demands imposed by these substrates on the tight reaction cavity of the Cu(II) reagent. On the other hand, α-substituted substrates (entries 2 and 3) afford overall yields (Me: 63% and Ph: 73%) that are very similar to those obtained with the Cu(I) analog. However, the higher acidity of the Cu(II)-derived manifold results in a significant number of products (enamine, 1,2-hydroxyamine, and imidazoline), which most likely arise from aziridine ring opening and further derivatization (β-hydrogen elimination, nucleophilic attack, and insertion of dipolarophile), as detailed in a previous publication [63].

Competitive aziridinations of equimolar amounts (4 equiv. each) of styrene versus an aliphatic olefin (1-hexene, allylbenzene, and 4-phenyl-1-butene; Table 4) using PhINTs (1 equiv.) in MeCN, mediated by the Cu(I) reagent (5 mol%), reveal chemoselectivities in favor of styrene by a large margin. These are significantly higher than those previously reported for [Cu(CH_3_CN)_4_](PF_6_) [72,73]. Unfortunately, the Cu(II) reagent afforded complex product profiles (possibly containing cross-coupling products) that did not permit evaluation of a trend in chemoselectivity.

**Figure 1:**
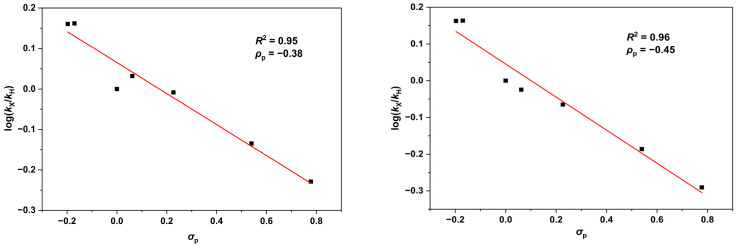
Linear free energy correlation of log(*k*_X_/*k*_H_) vs. *σ*_p_ for the aziridination of a panel of 4-X-styrenes (X = *^t^*Bu, Me, H, F, Cl, CF_3_, and NO_2_) mediated by [(^tBu^N4)Cu^I^(CH_3_CN)](PF_6_) (**left**) and [(^tBu^N4)Cu^II^(CH_3_CN)_2_](PF_6_)_2_ (**right**).

**Mechanistic Studies. (a) Hammett Plots.** To further unravel any differences in the operation of the Cu(I) and Cu(II) precatalysts, competitive aziridination experiments (PhINTs, 1 equiv.) were undertaken between styrene (4 equiv.) and a 4-X-substituted styrene (4 equiv.; X = *^t^*Bu, Me, Cl, F, CF_3_, and NO_2_) in MeCN at 25 °C, mediated by the Cu(I) or the Cu(II) reagent (5 mol%). The reaction was quenched after 3 h, and the ratio of aziridination products was quantified via ^1^H NMR (CD_3_CN) to provide *k*_X_/*k*_H_ data (Table S3). A satisfactory linear free energy correlation of log(*k*_X_/*k*_H_) as a function of the polar parameter *σ*_p_ is obtained (*ρ*_p_ = −0.38, *R*^2^ = 0.95) for reactions mediated by the Cu(I) precatalyst (Figure 1, left), which is only slightly improved by employing the resonance-sensitive parameter *σ*^+^ (*ρ*^+^ = −0.33, *R*^2^ = 0.96, Figure S2). In contrast to previous olefin aziridinations by Cu(I) reagents [71,74,75], application of Jiang’s dual parameter approach, involving both polar (*σ*_mb_) and spin-delocalization (*σ*_JJ_^•^) parameters [76], does not improve the correlation (*ρ*_mb_ = −0.35, *ρ*_JJ_^•^ = −0.23, *R*^2^ = 0.96, Figure S2), presumably due to the preponderance of polar over spin-delocalization effects (|*ρ*_mb_/*ρ*_JJ_^•^| = 1.52). For Cu(II)-mediated aziridinations, the polar parameter *σ*_p_ (*ρ*_p_ = −0.45, *R*^2^ = 0.96) affords a reasonable correlation (Figure 1, right). The *σ*^+^ scale and the dual-parameter approach (*σ*_mb_ and *σ*_JJ_^•^) provide inferior correlation levels. Only the *σ*_p_/*σ*_JJ_^•^ combination proved to be slightly better (Figure S2), denoting highly dominant polar over spin-delocalization effects (reportedly [76], |*ρ*_p_/*ρ*_JJ_^•^| values tend to be higher than |*ρ*_mb_/*ρ*_JJ_^•^| values). Notably, a recent report on the aziridination of fluorinated olefins by Cu(I) reagents also concluded that the *σ*_p_ scale is sufficient for obtaining excellent Hammett correlations [77]. These results indicate that a modest positive charge develops en route to the transition state, which is slightly more pronounced in aziridinations mediated by the Cu(II) precatalyst, potentially due to the high polarization of the resulting copper-nitrene bond (vide infra).

**Scheme 3:**
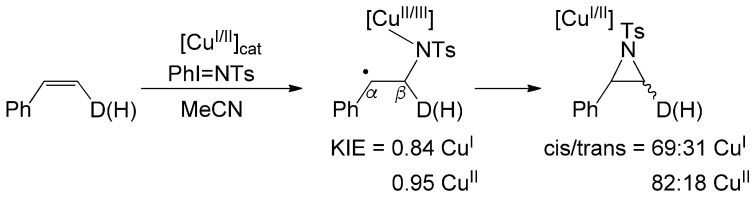
Putative copper nitrene addition to styrene and aziridine ring formation.

**(b) Deuterium Kinetic Isotope Effect.** To confirm whether the *β*-styrene position is the site of the initial metal-nitrene attack, we reacted equimolar amounts of styrene and cis-*β*-d_1_-styrene (1.0 mmol each) with PhINTs (0.125 mmol) in the presence of the Cu(I) or Cu(II) reagent (5 mol%) in acetonitrile at 25 °C. For the Cu(I) reagent, the ratio of protio/deuterio-containing aziridines was evaluated via ^1^H NMR to afford a KIE value of 0.84 (±0.02). The cis/trans ratio of aziridines was more accurately evaluated by means of ^2^H NMR, either from the same competitive experiment or by employing cis-*β*-d_1_-styrene as a single substrate. Modest levels of stereoretention (cis/trans = 69:31) were obtained for the Cu(I) precatalyst, consistent with previously reported values for other Cu(I) reagents from our lab [71]. A similar aziridination experiment with equimolar amounts of styrene and *α*-d_1_-styrene furnishes a KIE value close to unity (1.01 (±0.02)), thus suggesting that the initial attack of the metal nitrene is at the *β*-styrene position (Scheme 3).

The corresponding values for the Cu(II)-mediated aziridinations indicate a more modest inverse KIE (0.95 (±0.02)) with respect to the *β*-deuterated styrene site, accompanied by significantly better levels of stereoretention (cis/trans aziridine = 82:18). The KIE value for *α*-deuterated styrene is essentially 1 (1.02 (±0.02)).

Taken together, these results suggest that the Cu(II) precatalyst is associated with a relatively more reactant-resembling transition state (N^…^C*_β_* bond formation) and a faster aziridine ring-closure (N^…^C*_α_* bond formation) compared to the Cu(I) analog. The former may be a result of the steric requirements imposed by the tightly bound Cu(II) reagent. The latter is in agreement with previous observations [71] since the oxidative ring closure is facilitated by the ease of reduction of the metal site that is attached to the nitrene (as well as by the ease of carboradical oxidation) (Scheme 3). Two-step reactivity is anticipated for both Cu(I)- and Cu(II)-derived nitrenes, but the differentiation of the mechanistic parameters noted above suggests that distinct redox manifolds are operative.

Incidentally, the intermediate carboradical shown in Scheme 3 can be readily oxidized to the corresponding carbocation for suitable substrates (such as the α-substituted styrenes in Table 3, entries 2 and 3), and can in turn provide several other products, such as enamines (via β-hydrogen elimination), hydroxylamines (via nucleophilic attack), or imidazolines (via 3 + 2 insertion of MeCN). The incipient carbocation may also be the result of partial aziridine ring opening upon metal coordination (especially with the more acidic Cu(II)) [63].

### Computational Studies

Calculations were performed using the ORCA package (version 4.2.1) to probe the electronic structure of the putative copper-nitrene active species. Full experimental models were analyzed using density functional theory for both copper(I) and copper(II) precatalysts. Geometry optimizations were conducted at the B3LYP/def2-svp/CPCM-MeCN level of theory, with a single point calculation using the larger def2-tzvpp at the optimized geometry for property calculations, which were derived from a Mulliken population analysis. All geometry optimizations were initiated from crystal structures and invoked no symmetry constraints [78,79,80,81]. For these studies, the nitrene substituent is chosen to be p-toluenesulfonyl. Tables of coordinates for all spin states examined are provided as Supplementary Material.

Initially, the impact of acetonitrile coordination was assessed. The binding energy of acetonitrile to the five-coordinate complexes is ~−4 ± 1 kcal/mol, varying little with spin state or copper formal oxidation state. Adding unfavorable entropic effects for ligand coordination suggests that acetonitrile binding to the copper center is moderately exothermic but overall endergonic. Spin density and atomic charge values (vide infra) change little upon coordination of acetonitrile to the copper(I) center, so this discussion will largely focus on five-coordinate nitrene models.

**Figure 2:**
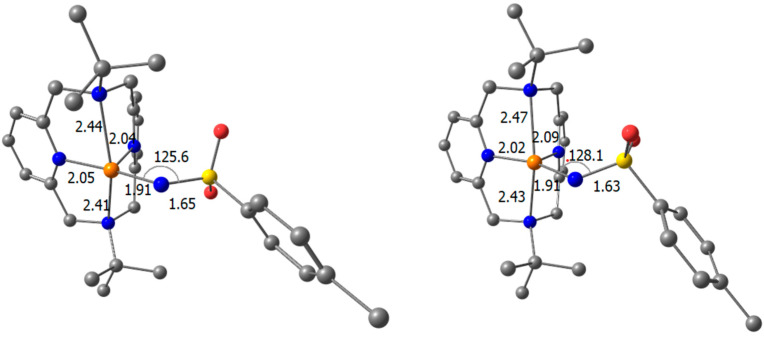
B3LYP/def2-svp/CPCM-MeCN optimized geometry of the copper(I)-derived five-coordinate Cu-nitrene. Triplet (**left**) and broken-symmetry singlet (**right**). Bond lengths in Angstrom units; angles in degrees. Hydrogens are omitted from the figure for clarity. N = blue, C = gray, O = red, S = yellow, and Cu = orange.

For the Cu(I)-derived copper-nitrene models, the triplet state is predicted to be slightly (~2 kcal/mol) lower in energy than the broken-symmetry (BS) singlet for both five-coordinate (Figure 2) and six-coordinate models. For singlet and triplet states, the spin density on the five-coordinate copper is computed via a Mulliken population analysis to be ~+½ e^−^; the spin density on the nitrenoid nitrogen is −0.7 e^−^ for the BS singlet and higher at + 1.0 e^−^ for the triplet. Atomic charges—again via a Mulliken analysis—are nearly identical for each spin state, ca. +½ e^−^ for copper and −½ e^−^ for the nitrenoid nitrogen.

**Figure 3:**
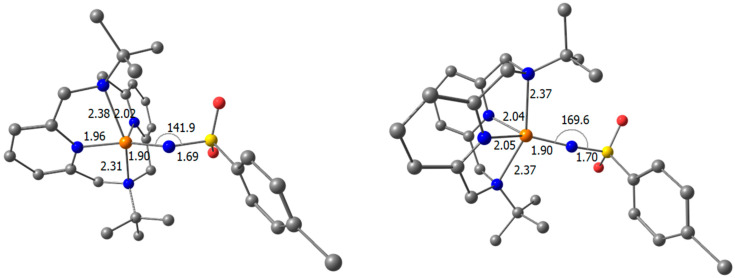
B3LYP/def2-svp/CPCM-MeCN-optimized geometry of copper(II)-derived five-coordinate Cu-nitrene. Doublet (**left**) and quartet (**right**). Bond lengths in Angstrom units; angles in degrees. Hydrogens are omitted from the figure for clarity. N = blue, C = gray, O = red, S = yellow, and Cu = orange.

For the copper(II)-derived models, the doublet and quartet states are essentially degenerate for the five-coordinate model (Figure 3), and upon coordination of acetonitrile, the lower spin state is predicted to be favored by a modest amount (3 kcal/mol). In the doublet state, the spin density on the five-coordinate copper is modest, ~−0.1 e^−^, while the spin density on the nitrenoid nitrogen is significant, +1.3 e^−^. The quartet state spin densities for the active site are +0.5 (Cu) and +1.5 e^−^ (nitrene N), with the remaining spin density diffused among the remaining atoms. Atomic charges in the doublet state are ca. +0.3 e^−^ for copper and −0.4 e^−^ for the nitrenoid nitrogen. The quartet state has a somewhat more polarized copper-nitrene bond than the corresponding doublet, with q(Cu) = +0.6 e^−^ and q(N) = −0.5 e^−^.

A general electrophilicity index (GEI) was also calculated using Stephan’s approach [82]. For the Cu(I)-derived copper nitrenes, both the triplet and broken-symmetry singlet states have a GEI value of 0.33 eV for the five-coordinate species, and essentially the same value for the six-coordinate analog (triplet: 0.34, BS singlet: 0.33 eV). For the Cu(II)-derived copper nitrenes, the doublet and quartet states for the five-coordinate species are computed to have a GEI value of 0.26 and 0.27 eV, respectively. The corresponding values for the six-coordinate congeners are 0.21 and 0.18 eV. Therefore, the Cu(I) precatalyst gives rise to a more electrophilic copper-nitrene moiety, in agreement with the higher reactivity of the Cu(I)-dependent manifold. The electrophilicity of the metal-nitrene (especially with nitrenes carrying electron-withdrawing substituents) has emerged as a primary criterion of reactivity in nitrene-transfer chemistry [20,83], although other secondary contributing factors may occasionally intervene [84].

## 3. Experimental Section

### 3.1. General Catalytic Olefin Aziridination Procedure

In a 20 mL screw-cap vial containing a small magnetic bar, the metal catalyst (0.0125 mmol), N-(*p*-tolylsulfonyl)imido-phenyliodinane (93.3 mg, 0.25 mmol), and molecular sieves (5Å) (20 mg) were weighed in sequence. Olefin (2.0 mmol) and solvent DCM (0.2 mL)/1,2-DCE (0.2 mL)/MeCN (0.3 mL) were charged in a separate vial. The olefin solution was then added to the vial containing the catalyst. The reaction mixture was stirred vigorously at 25 °C for 24 h (unless otherwise stated). After completion of the reaction, the products were purified via column chromatography (silica gel), first by eluting with hexanes (~30 mL) to remove the substrate, and then with petroleum ether/ethyl acetate (5:1 *v*/*v*, ~60 mL) to remove the catalyst and elute product(s). Yields were quantified via ^1^H NMR (in CDCl_3_) versus an internal standard (4-methoxyacetophenone) (i.e., all reported yields are NMR yields). All aziridination and amination products (Table 1, Table 2, Table 3 and Table 4) are known compounds and were identified with the assistance of ^1^H and ^13^C NMR spectra, through comparison with spectroscopic features reported for authentic samples in the literature [63,64,71,72,84,85,86].

### 3.2. General Chemo-Selective Reaction Procedure

For the competitive aziridinations of styrene versus 1-hexene, allylbenzene, and 4-phenyl-1-butene (Table 4), mediated by [(^tBu^N4)Cu^I^(CH_3_CN)](PF_6_) (or [(^tBu^N4)Cu^II^(CH_3_CN)_2_](PF_6_)_2_), the general aziridination procedure noted above was followed, with the exception that 1.0 mmol of each olefin was used in acetonitrile.

### 3.3. Catalytic Reaction Procedure for Hammett Analysis

For each competition experiment, a 20 mL screw-cap vial was charged in sequence with the catalyst [(^tBu^N4)Cu^I^(CH_3_CN)](PF_6_) or [(^tBu^N4)Cu^II^(CH_3_CN)_2_](PF_6_)_2_ (5 mol%), N-(*p*-tolylsulfonyl)imido-phenyliodinane (46.6 mg, 0.125 mmol), molecular sieves (5Å) (20 mg), and a small magnetic bar. Styrene (1.0 mmol), *p*-X-styrene (1.0 mmol, X = Me, *^t^*Bu, F, Cl, CF_3_, and NO_2_), and acetonitrile (0.30 mL) were mixed in a separate vial. The olefin solution was then added to the vial containing the solids, and the reaction mixture was stirred vigorously at 25 °C for 3 h. After this period, the aziridination products were purified via column chromatography, first by eluting with hexanes (~35 mL) to remove olefins and then with petroleum ether/ethyl acetate (8/1 *v*/*v*, ~50 mL) to collect the aziridines. The product ratio (*k*_X_/*k*_H_) was calculated via quantitative ^1^H NMR analysis in CD_3_CN (relaxation delay: 120 s).

### 3.4. Competitive Aziridinations of Deuterated Styrenes vs. Styrene (Evaluation of KIE)

In order to determine the values of secondary deuterium kinetic isotope effects (*k*_H_/*k*_D_), a diagnostic deuterated styrene (*a*-d_1_-styrene or *cis*-*b*-d_1_-styrene) was used together with styrene (1.0 mmol each) and was subjected to aziridination using PhI=NTs (46.6 mg, 0.125 mmol) in the presence of [(^tBu^N4)Cu^I^(CH_3_CN)](PF_6_) or [(^tBu^N4)Cu^II^(CH_3_CN)_2_](PF_6_)_2_ (5 mol%) and molecular sieves (5 Å, 20 mg) in MeCN (0.30 mL), according to the general olefin aziridination procedure noted above. Prior to mixing with any other reagents, a drop of the original mixture of deuterio and protio styrene was retained for ^1^H-NMR analysis (CDCl_3_, 150 s relaxation delay) to determine the starting d_0_/d_1_ styrene ratio. The reaction was quenched after 0.5 h, and the mixture was purified using flash chromatography on silica gel as noted above (Section 3.3) to recover the aziridination products. In order to extract secondary KIE values, the ratio of d_0_/d_1_ aziridines is evaluated using ^1^H NMR (CD_3_CN, 120 s relaxation delay) and compared to the ratio of the starting d_0_/d_1_ styrenes.

### 3.5. Stereochemical Scrambling in the Aziridination of cis-b-d_1_-Styrene

The aziridination of *cis*-*b*-d_1_-styrene, mediated by [(^tBu^N4)Cu^I^(CH_3_CN)](PF_6_) or [(^tBu^N4)Cu^II^(CH_3_CN)_2_](PF_6_)_2_ (5 mol%), was conducted according to the general procedure for olefin aziridinations. The purified aziridines were dissolved in CHCl_3_ (70 µL)/CDCl_3_ (2 µL) and loaded into a precision NMR tube. ^2^H NMR data were collected (relaxation delay: 15 s) from two independent reaction trials to quantify the relative deuterium content at the cis and trans *β*-carbon sites of the aziridination products and thus determine the cis/trans aziridination ratio.

Additional experimental procedures and general information can be found in the Supplementary Materials (vide infra).

## 4. Conclusions

The following are the major findings and insights gained from the present study:

(i) A series of divalent compounds [(^tBu^N4)M^II^(CH_3_CN)_2_](PF_6_)_2_ (M = Mn, Fe, Co, Ni, and Cu) and monovalent [(^tBu^N4)Cu^I^(CH_3_CN)](PF_6_) were synthesized with the known 12-membered Py_2_N_2_-type pyridinophane macrocycle ^tBu^N4 and hexafluorophosphate as counter anion, for catalytic and mechanistic evaluation as mediators of olefin aziridinations via nitrene-transfer chemistry. The Mn^II^ and Cu^I^ congeners had been previously synthesized.
(ii) For the aziridination of styrene and para-substituted styrenes using PhI=NTs, the Cu(I) reagent proved to be the most effective catalyst, with yields exceeding 80%, even for electron-withdrawing para substituents. The Cu(II) precatalyst is also highly competitive, with the exception of strongly electron-withdrawing para substituents (e.g., CF_3_ and NO_2_), for which yields drop to approximately 60%. All other divalent reagents mediate the same styrene aziridinations with modest yields (not exceeding 50%), demonstrating an approximate trend of Fe ≥ Mn ≥ Ni ≈ Co. As noted previously [63], these dicationic base metals give rise to metal-nitrene moieties that tend to decompose due to high electron deficiency, affording high TsNH_2_ yields.
(iii) The best behaving Cu(I) and Cu(II) precatalysts also afford practicable aziridination yields with other styrenes, such electron-rich α-substituted congeners. However, bulky ortho- and/or *β*-substitution hamper yields, affecting turnover more prominently with the Cu(II) catalyst, presumably due to its less voluminous reaction cavity. Allylic aminations and other byproducts of aziridine-ring formation or cleavage are also observed, especially in chlorinated solvents that raise the acidity of the metal sites. As expected, the aziridination of aliphatic-substituted olefins is generally low-yielding. Notably, competitive aziridination of styrene vs. 1-hexene favors the former by a wide margin (27:1 for Cu(I)).
(iv) Although Cu(I) or Cu(II) precatalysts have been shown to occasionally operate via the same catalytic cycle [73], the present study suggests that [(^tBu^N4)Cu^I^]^+^ and [(^tBu^N4)Cu^II^]^2+^ work via different catalytic manifolds. First, the Cu(I) congener mediates aziridination of unencumbered substrates, such as styrene, much faster than the Cu(II) analog. Although this may be due to the slower reaction of PhI=NTs with the Cu(II) site, other mechanistic indicators suggest that the Cu(I) and Cu(II) precatalysts may operate via distinct pathways. Hammett analysis and KIE parameters indicate that both precatalysts follow the general two-step mechanistic path (successive formation of N–C*_β_* and N–C*_α_* bonds in the aziridination of styrene), but the Cu(I) catalyst is associated with a more modest positive charge at the styrenyl *α*-carbon as well as a larger inverse KIE value in the process of formation of the first N–C*_β_* bond, suggesting a more carboradical intermediate-like transition state. On the other hand, the Cu(II)-related manifold exhibits superior stereochemical integrity associated with the fast closure of the second, product-determining N–C*_α_* bond.
(v) Computational studies reveal that the triplet state of the putative five-coordinate [Cu] = NTs moiety (devoid of MeCN binding), generated from the Cu(I) precatalyst, is slightly (~2 kcal/mol) lower in energy than the broken-symmetry (BS) singlet. For the corresponding Cu(II) derived nitrene, the doublet and quartet states are almost degenerate. In all cases, significant spin density is localized on the nitrene N atom. Dual spin-state reactivity is thus possible for both mechanistic paths and may further complicate mechanistic analysis. Importantly, the fast-operating Cu(I)-mediated manifold is consistent with the superior electrophilicity of the Cu(I)-derived nitrene, as computed for all spin states.

Overall, among all catalysts explored in the present study, the more rapid [(^tBu^N4)Cu^I^]^+^ and the more product-selective [(^tBu^N4)Cu^II^]^2+^ constitute viable nitrene-transfer reagents for the aziridination of unencumbered aromatic olefins. We conclude that further experimentation with stereogenic substituents in lieu of *^t^*Bu is warranted, in order to identify suitable catalysts for highly selective (including enantioselective) nitrene-transfer chemistry in high demand for simple olefins.

## Figures and Tables

**Table 1:** Yields of aziridination of para-substituted styrenes mediated by various metal complexes *^a^*.

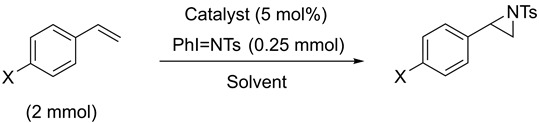							
Entry No.	Product	^tBu^N4Mn^II^ Yield (%)	^tBu^N4Fe^II^ Yield (%)	^tBu^N4Co^II^ Yield (%)	^tBu^N4Ni^II^ Yield (%)	^tBu^N4Cu^II^ Yield (%)	^tBu^N4Cu^I^ Yield (%)
1.	X = H	39	40	32	37	86	91
2.	X = Me	23	40	22	28	79	89
3.	X = ^t^Bu	30	47	33	28	86	91
4.	X = Cl	38	47	32	27	97	92
5.	X = F	30	46	28	25	98	84
6.	X = CF_3_	23	36	20	16	57	82
7.	X = NO_2_	28	48	11	11	63	89

*^a^* M(I/II) catalyst, 0.0125 mmol (5 mol%); PhINTs, 0.25 mmol; olefin, 2.0 mmol; MS 5 Å, 20 mg; solvent (MeCN), 0.30 mL; 25 °C; 12 h; NMR yields.

**Table 2:** Yields of aziridination of para-substituted styrenes mediated by [(^tBu^N4)Cu^I^(CH_3_CN)](PF_6_) *^a^*.

Entry No.	Substrate	Product		DCM Yield (%)	1,2-DCE Yield (%)	MeCN Yield (%)
1.	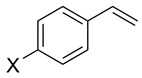	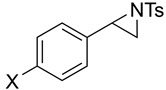	X = H	88	96	91
2.			X = Me	85	90	89
3.			X = ^t^Bu	94	91	91
4.			X = OMe	Polymer	Polymer	57
5.			X = O^t^Bu	Polymer	Polymer	77
6.			X = Cl	92	99	92
7.			X = F	93	82	84
8.			X = CF_3_	90	99	82
9.			X = NO_2_	98	99	89

*^a^* [(^tBu^N4)Cu^I^(MeCN)](PF_6_), 0.0125 mmol (5 mol%); PhINTs, 0.25 mmol; olefin, 2.0 mmol; MS 5 Å, 20 mg; solvent, 0.2 mL (DCM and 1,2-DCE), 0.3 mL (MeCN); 25 °C; 12 h; NMR yields.

**Table 3:** Yields of aziridination and amination products of various olefins mediated by [(^tBu^N4)Cu^I^(CH_3_CN)](PF_6_) in various solvents *^a^* (or [(^tBu^N4)Cu^II^(CH_3_CN)_2_](PF_6_)_2_ in MeCN for selected substrates; yields in parenthesis *^b^*).

Entry No.	Substrate	Products		DCM Yield (%)	1,2-DCE Yield (%)	MeCN Yield (%)
1.	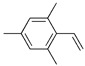	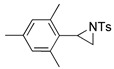		49	44	42 (30) *^b^*
2.	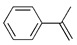	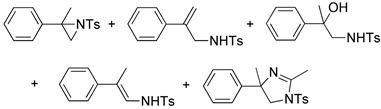		24, 9, 19	23, 8, 29	42, 5, 16, 4, 0 (28, 6, 12, 0, 17) *^b^*
3.	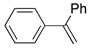	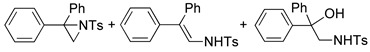		9, 50, 9	2, 52, 12	30, 30, 12 (2, 49, 22) *^b^*
4.	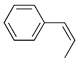	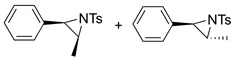		21, 27	24, 23	28, 29 (13, 11) *^b^*
5.	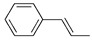	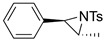		37	32	29 (18) *^b^*
6.	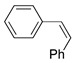	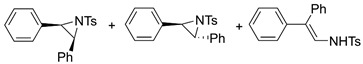		8, 26, 3	9, 26	14, 28 (6, 3, 9) *^b^*
7.	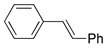	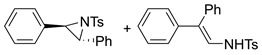		17, tr	12, tr	19 (5, tr) *^b^*
8.	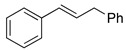	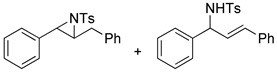		10, 32	11, 33	15, 26
9.	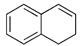	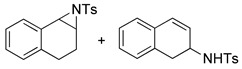		24, tr	26, tr	27, tr
10.	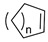	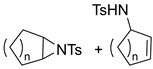	*n* = 1	4, 8	4, 12	4, 16
11.			*n* = 2	4, 10	6, 12	6, 2
12.			*n* = 3	22	30	29
13.	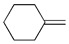	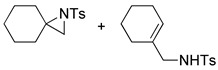		22, 6	15, 2	30, 3
14	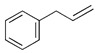	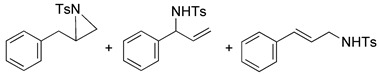		10, 8, 8	8, 7, 8	8, 2, 3
15	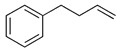	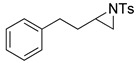		22	25	21
16	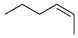	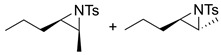		11, 4	11, 4	14, 3
17.	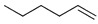	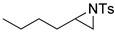		10	10	11
18.	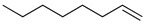	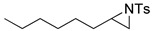		9	8	13

*^a^* Cu(I) or Cu(II) catalyst, 0.0125 mmol (5 mol %); PhINTs, 0.25 mmol; olefin, 2.0 mmol; MS 5 Å, 20 mg; solvent, 0.2 mL (DCM and 1,2-DCE), 0.3 mL (MeCN); 25 °C; 24 h. *^b^* Yields for Cu(II) precatalyst; NMR yields.

**Table 4:** Competitive aziridination of styrene vs. 1-hexene, allylbenzene, and 4-phenyl-1-butene.

Catalyst	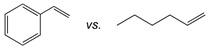	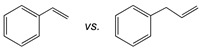	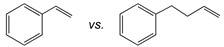
[(^tBu^N4)Cu^I^(MeCN)](PF_6_)	27:1	21:1	18:1

## Data Availability

The original contributions presented in this study are included in the article. Further inquiries can be directed to the corresponding authors.

## Supplementary Materials

The following supporting information can be downloaded at https://www.mdpi.com/article/10.3390/molecules30153097/s1: additional synthetic protocols; physicochemical characterization of new compounds; X-ray crystallographic and computational data; additional figures and tables as noted in the text. References [87,88,89,90,91] are cited in Supplementary Materials.
