# Supplementary material for: Comparative Nitrene-Transfer Chemistry to Olefins Mediated by First-Row Transition Metal Catalysts Supported by a Pyridinophane Macrocycle with N4 Ligation
Source: Molecules. 2025 Jul 24;30(15):3097. doi: 10.3390/molecules30153097 (PMC12348876; doi:10.3390/molecules30153097)
Supplement: Supplementary file 1 [file molecules-30-03097-s001.zip › molecules-3717607-supplementary.pdf]

Comparative Nitrene-Transfer Chemistry to Olefins Mediated by First-Row Transition Metal Catalysts Supported by a Pyridinophane Macrocycle with N4 Ligation

Himanshu Bhatia [1], Lillian P. Adams [1], Ingrid Cordsiemon [1], Suraj Kumar Sahoo [1], Amitava Choudhury [1], Thomas R. Cundari [2,*] and Pericles Stavropoulos [1,*]

[1] Department of Chemistry, Missouri University of Science and Technology, Rolla, MO 65409, USA; hbgxg@umsystem.edu (H.B.); lillian.adams.1@slu.edu (L.P.A.); iccord3@gmail.com (I.C.); surajsahoo2392@gmail.com (S.K.S.); choudhurya@mst.edu (A.C.)

[2] Department of Chemistry, Center for Advanced Scientific Computing and Modelling (CASCaM), University of North Texas, Denton, TX 76203, USA

* Correspondence: thomas.cundari@unt.edu (T.R.C.); pericles@mst.edu (P.S.); Tel.: +1-573-341-7220 (P.S.); Fax: +1-573-341-6033 (P.S.)

Table of Contents

Experimental Section......S3

General Considerations......S3

Synthesis of Ligand......S4

Safety warning......S4

Scheme S1. Synthesis of ligand......S4

Synthesis of Metal Compounds......S6

Scheme S2. Synthesis of metal compounds......S6

Other physical measurements......S11

X-ray crystallography......S11

Table S1. Summary of Crystallographic Data for [(^{tBu}N4)Fe(MeCN)$_2$](PF$_6$)$_2$, [(^{tBu}N4)Co(MeCN)$_2$](PF$_6$)$_2$, [(^{tBu}N4)Ni(MeCN)$_2$](PF$_6$)$_2$ and [(^{tBu}N4)Cu(MeCN)$_2$](PF$_6$)$_2$......S12

Figure S1. ORTEP Diagrams of [(^{tBu}N4)Fe(MeCN)$_2$](PF$_6$)$_2$, [(^{tBu}N4)Co(MeCN)$_2$](PF$_6$)$_2$, [(^{tBu}N4)Ni(MeCN)$_2$](PF$_6$)$_2$, [(^{tBu}N4)Cu(MeCN)$_2$](PF$_6$)$_2$ (cations only) drawn with 40% thermal ellipsoids......S13

Table S2. Selective Bond Lengths (Å) and Bond Angles (°) of Metal Compounds......S14

Mechanistic Studies......S15

Table S3. Competitive Aziridination Reactions of 4-X-Styrenes *vs.* Styrene by PhI=NTs in the presence of [(^{tBu}N4)Cu^{I}(MeCN)](PF$_6$) or [(^{tBu}N4)Cu^{II}(MeCN)$_2$](PF$_6$)$_2$^{a}......S15

Figure S2. Linear free energy correlation of log(k_X/k_H) as a function of σ^+ for the competitive aziridination of 4-X-styrene vs styrene catalyzed by [(^{tBu}N4)Cu^{I}(MeCN)](PF$_6$) (top); log(k_X/k_H) as a function of σ_{mb} and σ_{jj}• for the competitive aziridination of 4-X-styrene vs styrene catalyzed by [(^{tBu}N4)Cu^{I}(MeCN)](PF$_6$) (middle); and log(k_X/k_H) as a function of σ_p and σ_{jj}• for the competitive aziridination of 4-X-styrene vs styrene catalyzed by [(^{tBu}N4)Cu^{II}(MeCN)$_2$](PF$_6$)$_2$ (bottom)......S16

Representative Mechanistic NMR Data......S17

Computational Data......S20

References......S33

EXPERIMENTAL SECTION

General Considerations. All operations were performed under anaerobic conditions under a pure dinitrogen atmosphere using Schlenk techniques on an inert gas/vacuum manifold or in a dry box (O_2, H_2O < 1 ppm). Anhydrous diethyl ether, methylene chloride, 1,2-dichloroethane, acetonitrile, hexane, pentane, benzene, toluene, dimethylformamide, dimethylacetamide, and dimethyl sulfoxide were purchased from Sigma-Aldrich (Millipore). Solvents were degassed via three freeze–pump–thaw cycles. Unless otherwise noted, all other reagents were purchased at the highest available purity or synthesized according to literature procedures noted in the text.

^1H and ^{13}C NMR spectra were recorded on Varian XL-400, Varian INOVA/ UNITY 400 MHz Unity Plus, and Varian 300 Unity Plus NMR spectrometers. IR spectra were obtained on a Perkin-Elmer 883 IR spectrometer, and FT-IR spectra on Nicolet Nexus 470 and 670, Magna 750 FT-IR ESP, and Shimadzu IR-Affinity-1 spectrometers. UV–vis spectra were obtained on a Hewlett-Packard 8452A diode array, Varian Cary 50, and Varian Cary 300 spectrophotometers. HRMS data were collected on a Thermo Fisher Scientific LTQ-Orbitrap XL hybrid mass spectrometer, using the Orbitrap analyzer for acquisition of high-resolution, accurate mass data. Samples were infused using the integrated syringe pump at 3 μL/min, and ionization was performed via the electrospray source with source settings at their defaults. In general, settings for the ion optics were determined automatically during the regular tuning and calibration of the instrument. For high-resolution data, the Orbitrap analyzer was set to a resolution of 100000. Microanalyses were conducted on an in-house Perkin-Elmer 2400 CHN analyser. Other physical measurements are noted below.

Synthesis of Ligand

Safety Warning

Benzene is a known carcinogen and can cause various health problems. All processes using benzene should be performed in a fume hood. Personal protective equipment (PPE) should be worn. Thallium compounds are highly toxic and must be handled with extreme care.

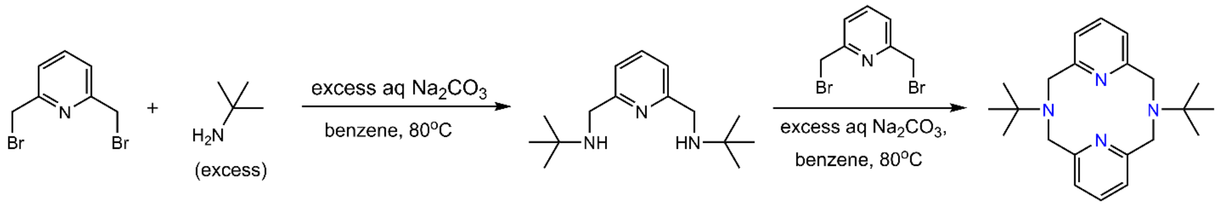

Scheme S1. Synthesis of ligand (^{tBu}N4).

2,6-Bis[N-(t-butylamino)methyl]pyridine

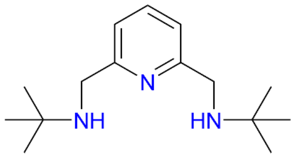

This compound was prepared according to reported procedures with slight modifications.[1] To a mixture of *tert*-butylamine (13.3 g, 181.2 mmol) in 15% aqueous sodium carbonate (40 mL), a solution of 2,6-bis(bromomethyl)pyridine (1.6 g, 6.039 mmol) in benzene (60 mL) was added using a dropping funnel attached to a multi-necked flask over the course of 30 minutes, with continuous stirring. The reaction mixture was refluxed for 8 hours. The mixture was then cooled

to room temperature, and the organic phase was separated. The aqueous layer was again extracted with benzene (2 × 100 mL). The combined organic layers were dried over $MgSO_4$ and Na_2CO_3, and then benzene and unreacted amine were removed under vacuum, affording the desired product as an oily yellow substance (1.28 g, 85% yield). ^{1}H NMR ($CDCl_3$, 7.26 ppm): δ 1.18 (18 H, s, CH_3), 1.82 (2H, br, NH), 3.85 (4H, s, $PyCH_2$), 7.16 (2H, d, 3,5- PyH), 7.55 (1H, t, 4-PyH). ^{13}C NMR ($CDCl_3$, 77.16 ppm): δ 29.3, 48.6, 50.6, 120.3, 136.9, 159.9.

N,N′-Di-tert-butyl-2,11-diaza[3.3](2,6)pyridinophane (^{tBu}N4)

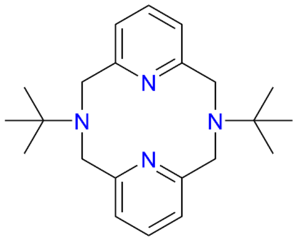

(^{tBu}N4)

2,6-Bis[N-(*t*-butylamino)-methyl]pyridine (1.64 g, 6.56 mmol) was dissolved in 5.0 mL benzene and added to a 15% aqueous sodium carbonate solution (40 mL). To the former mixture, 2,6-bis(bromomethyl)pyridine (1.58 g, 5.96 mmol) dissolved in 60 mL of benzene was added dropwise over 60 minutes via an addition funnel. The reaction mixture was allowed to reflux for 10 hours. Saturated Na_2CO_3 solution was added to this mixture to avoid any deprotonation of the ligand. The mixture was then cooled down, and the organic phase was separated. The aqueous layer was again extracted with benzene (3 × 150 mL). The combined organic layers were dried over $MgSO_4$ and Na_2CO_3, and the benzene was removed under vacuum, affording a white solid, which was washed with heptane to afford a pure white solid. The white solid was dissolved in ethanol (150 mL) at 70 ºC for an hour. The mixture was cooled down, and white residue was removed via filtration, and the solution was evaporated to obtain the desired product (1.1 g,

52.3%). ¹H NMR (CDCl3, 7.26 ppm): δ 1.32 (18H, s, CH_3), 3.97 (8H, s, $PyCH_2$), 6.72 (4H, d, 3,5-Py H), 7.07 (2H, m, 4-Py H). ¹³C NMR (CDCl3, 77.16 ppm): δ 27.8, 56.0, 57.8, 122.0, 135.3, 159.5.

Synthesis of Metal Compounds

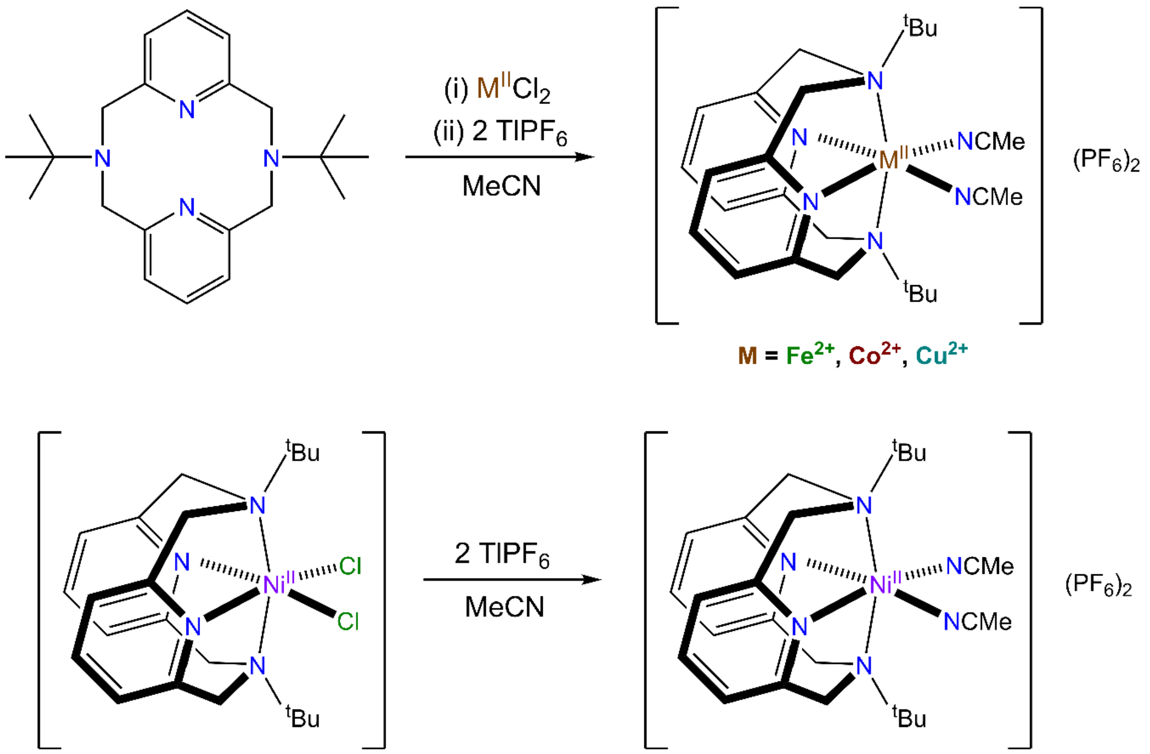

Scheme S2. Synthesis of metal compounds.

[(^{tBu}N4)Fe(MeCN)$_2$](PF$_6$)$_2$

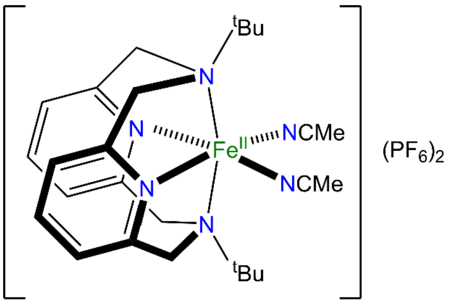

A solution of $TlPF_6$ (268.6 mg, 0.768 mmol) in 8.0 mL of MeCN was added to a pre-stirred (10 h) yellow suspension of ^{t}BuN4 (145.4 mg, 0.413 mmol) and $FeCl_2$ (47.6 mg, 0.375 mmol) in 5.0 mL of MeCN. Upon completion of the $TlPF_6$ addition, white TlCl precipitated, accompanied by a solution color change from yellow to green. The reaction mixture was stirred at room temperature for 6 hours in the dark, then left to stand without stirring for 30 min. The reaction mixture was subsequently filtered through an anaerobic frit. The solution was then reduced to 4.0 mL under vacuum, and diethyl ether (35.0 mL) was carefully layered over the MeCN solution, which was allowed to mix slowly at −35 ºC to afford crystalline green material (207.8 mg, 71%). IR (KBr, cm^{-1}) 3636, 2973, 2280, 1980, 1604, 1582, 1476, 1439, 1406, 1381, 1262, 1229, 1184, 1075, 1031, 939, 877, 828, 787, 778, 740, 712, 555. UV-Vis (MeCN): λ (ε (M^{-1} cm^{-1})) 354 (1777), 396 (1908), 668 (25). Elem. Anal. calcd. for $C_{26}H_{38}N_6FeF_{12}P_2$: C, 40.02; H, 4.91; N, 10.77. Found C, 40.13; H, 4.92; N, 10.74.

[(^{tBu}N4)Co(MeCN)$_2$](PF$_6$)$_2$

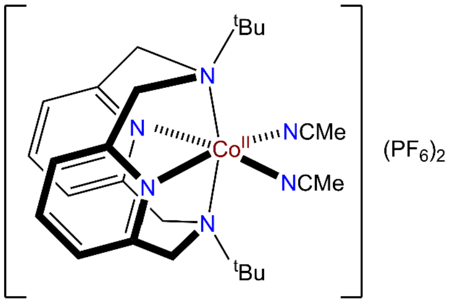

A solution of $TlPF_6$ (247.8 mg, 0.709 mmol) in 7.0 mL of MeCN was added to a pre-stirred (10 h) blue suspension of ^{tBu}N4 (134.1 mg, 0.380 mmol) and $CoCl_2$ (44.9 mg, 0.346 mmol) in 6.0 mL of MeCN. Upon completion of the $TlPF_6$ addition, white TlCl precipitated, with a solution color change from blue to orange. The reaction mixture was stirred at room temperature for 6 hours in the dark and then left to stand without stirring for 30 min. The reaction mixture was then filtered through an anaerobic frit. The solution was subsequently reduced to 4.0 mL under vacuum, and diethyl ether (35.0 mL) was carefully layered over the MeCN solution and allowed to mix slowly at −35 °C to afford crystalline orange material (115 mg, 42%). IR (KBr, cm^{-1}) 3612, 2980, 2325, 2297, 1980, 1606, 1581, 1471, 1438, 1403, 1384, 1260, 1229, 1191, 1168, 1085, 1043, 940, 918, 877, 825, 790, 740, 555. UV-Vis (MeCN): λ (ε (M^{-1} cm^{-1})) 308 (1437), 340 (934), 420 (54), 500 (32). Elem. Anal. calcd. for $C_{26}H_{38}N_6CoF_{12}P_2$: C, 39.86; H, 4.89; N, 10.73. Found C, 39.91; H, 4.90; N, 10.71.

[(^{tBu}N4)Ni(MeCN)$_2$](PF$_6$)$_2$.

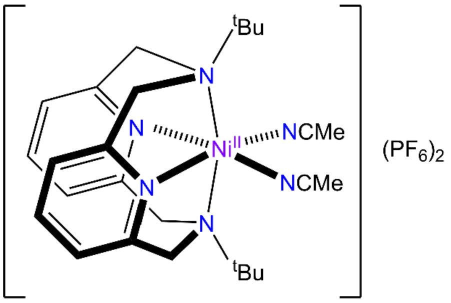

A solution of $TlPF_6$ (348 mg, 0.995 mmol) in 6.0 mL of MeCN was added to a pre-stirred (10 h) green solution of (^{tBu}N4)$NiCl_2$ (234 mg, 0.485 mmol) in 4.0 mL of MeCN. Upon completion of the $TlPF_6$ addition, white TlCl precipitated, accompanied by a solution color change from green to lilac. The reaction mixture was stirred at room temperature for 6 hours in the dark, then left to stand without stirring for 30 min. The reaction mixture was filtered through an anaerobic frit. The solution was then reduced to 3.0 mL under vacuum, and diethyl ether (35.0 mL) was carefully layered over the MeCN solution and allowed to mix slowly at −35 °C to afford crystalline light-purple material (248.5 mg, 65.4%). IR (KBr, cm^{-1}) 3642, 2948, 2303, 2286, 2050, 1605, 1584, 1470, 1439, 1406, 1379, 1260, 1187, 1074, 1040, 940, 910, 827, 790, 740, 555. UV-Vis (MeCN): λ (*ε* (M^{-1} cm^{-1})) 340 (100), 514 (8), 755 (7). Elem. Anal. calcd. for $C_{26}H_{38}N_6NiF_{12}P_2$: C, 39.87; H, 4.89; N, 10.73. Found C, 39.89; H, 4.90; N, 10.72.

[(^{tBu}N4)Cu(CH3CN)2](PF6)2

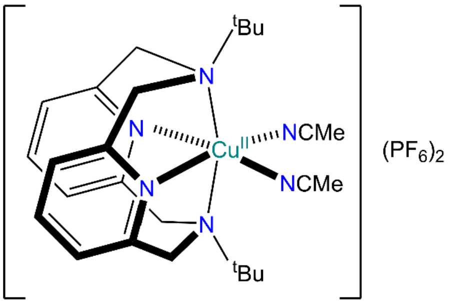

A solution of $TlPF_6$ (373.48 mg, 1.068 mmol) in 8.0 mL of MeCN was added to a pre-stirred (10 h) green suspension of ^{t}BuN4 (202 mg, 0.573 mmol) and $CuCl_2$ (70.1 mg, 0.521 mmol) in 5.0 mL of MeCN. Upon completion of the $TlPF_6$ addition, white TlCl precipitated, with a solution color change from green to teal. The reaction mixture was stirred at room temperature for 8 hours in the dark, then left to stand without stirring for 30 min. The reaction mixture was filtered through an anaerobic frit. The solution was then reduced to 4.0 mL under vacuum, and diethyl ether (35.0 mL) was carefully layered over the MeCN solution and allowed to mix slowly at −35 °C to afford crystalline green material (248 mg, 60.4 %). IR (KBr, cm^{-1}) 3564, 2979, 2322, 2294, 2103, 1990, 1604, 1581, 1468, 1437, 1403, 1386, 1369, 1261, 1225, 1194, 1167, 1086, 1028, 938, 922, 828, 790, 778, 740, 556. UV-Vis (MeCN): λ (ε (M^{-1} cm^{-1})) 372 (206), 640 (42). Elem. Anal. calcd. for $C_{26}H_{38}N_6CuF_{12}P_2$: C, 39.63; H, 4.86; N, 10.66. Found C, 39.68; H, 4.88; N, 10.64.

[(^{tBu}N4)Mn(MeCN)2](PF6)2 and [(^{tBu}N4)Cu(MeCN)](PF6) were prepared using literature procedures.[2,3]

Other Physical Measurements

X-ray Crystallography. Intensity data sets for all the compounds were collected on either a Bruker Smart Apex or a Bruker Apex II diffractometer using graphite-monochromated Mo (λ = 0.71073 Å) Kα radiation from a fine-focus sealed tube X-ray source. Suitable crystals were selected and mounted on a glass fiber using super glue. Datasets were collected at low temperatures ranging between 123 and 220 K (Table S1), employing a scan of 0.3° in ω with an exposure time of 20 s/frame using Apex III or SMART software.[4,5] Unit cell refinement and data reduction were carried out with SAINT, while the program SADABS was used for the absorption correction.[6] The structures were solved via direct methods using SHELXS-97 and difference Fourier syntheses.[7] Full-matrix least-squares refinement against $|F^2|$ was carried out using the SHELXTL-PLUS[6] suite of programs. All non-hydrogen atoms were refined anisotropically. Hydrogen atoms were placed geometrically and held in the riding mode during the final refinement. All structures were finally refined with SHELX-2014 using SHELXle.[8] The overall data quality for $[(^{tBu}N4)Fe(MeCN)_2](PF_6)_2$ and $[(^{tBu}N4)Ni(MeCN)_2](PF_6)_2$ was poor due to thin, needle-shape crystals, which resulted in B-level alerts. Explanations for the B-level alerts are provided in the validation response form (VRF) embedded in the CIF files. Unit cell parameters and pertinent crystallographic details are listed in Table S1. ORTEP-type diagrams and selective metrical parameters for all compounds are shown in Figure S1 and Table S2. The hexafluorophosphate (PF_6^-) counterions are not shown in the ORTEP-type diagrams.

Table S1. Summary of crystallographic data for $[(^{tBu}N4)Fe(MeCN)_2](PF_6)_2$, $[(^{tBu}N4)Co(MeCN)_2](PF_6)_2$, $[(^{tBu}N4)Ni(MeCN)_2](PF_6)_2$, and $[(^{tBu}N4)Cu(MeCN)_2](PF_6)_2$.

	$[(^{tBu}N4)Fe(MeCN)_2]$ $(PF_6)_2$	$[(^{tBu}N4)Co(MeCN)_2]$ $(PF_6)_2$	$[(^{tBu}N4)Ni(MeCN)_2]$ $(PF_6)_2$	$[(^{tBu}N4)Cu(MeCN)_2]$ $(PF_6)_2$
formula	$C_{26}H_{38}N_6FeP_2F_{12}$	$C_{26}H_{38}N_6CoP_2F_{12}$	$C_{26}H_{38}N_6NiP_2F_{12}$	$C_{26}H_{38}N_6CuP_2F_{12}$
M_r	780.41	783.49	783.25	788.10
crystal system	Monoclinic	Monoclinic	Monoclinic	Monoclinic
space group	$P2_1$	$P2_1/n$	$P2_1$	$P2_1/n$
a (Å)	17.421(9)	11.110(2)	9.083(3)	12.6384(5)
b (Å)	9.064(4)	16.580(2)	17.579(6)	16.3350(6)
c (Å)	20.033(9)	18.103(2)	20.585(7)	15.9202(6)
α (deg)	90	90	90	90
β (deg)	90.043(9)	90.935(2)	90.009(6)	93.821(2)
γ (deg)	90	90	90	90
V (Å^3)	3163(3)	3334.4(8)	3287(2)	3279.4(2)
Z	4	4	4	4
D_{calcd} (g cm^{-3})	1.639	1.561	1.583	1.596
T (K)	123(2)	220(2)	200(2)	173(2)
λ (Å)	0.71073	0.71073	0.71073	0.71073
μ (mm^{-1})	0.679	0.706	0.982	0.861
$R_1{}^a$ (I>2sigma(I))	0.0831	0.0722	0.0791	0.0391
$wR_2{}^b$ (I>2sigma(I))	0.1831	0.1885	0.1062	0.1045

^{a} $R_1 = \Sigma||F_o| - |F_c||/\Sigma|F_o|$. ^{b} $wR_2 = [\Sigma w(F_o{}^2 - F_c{}^2)^2/\Sigma w(F_o{}^2)^2]^{1/2}$

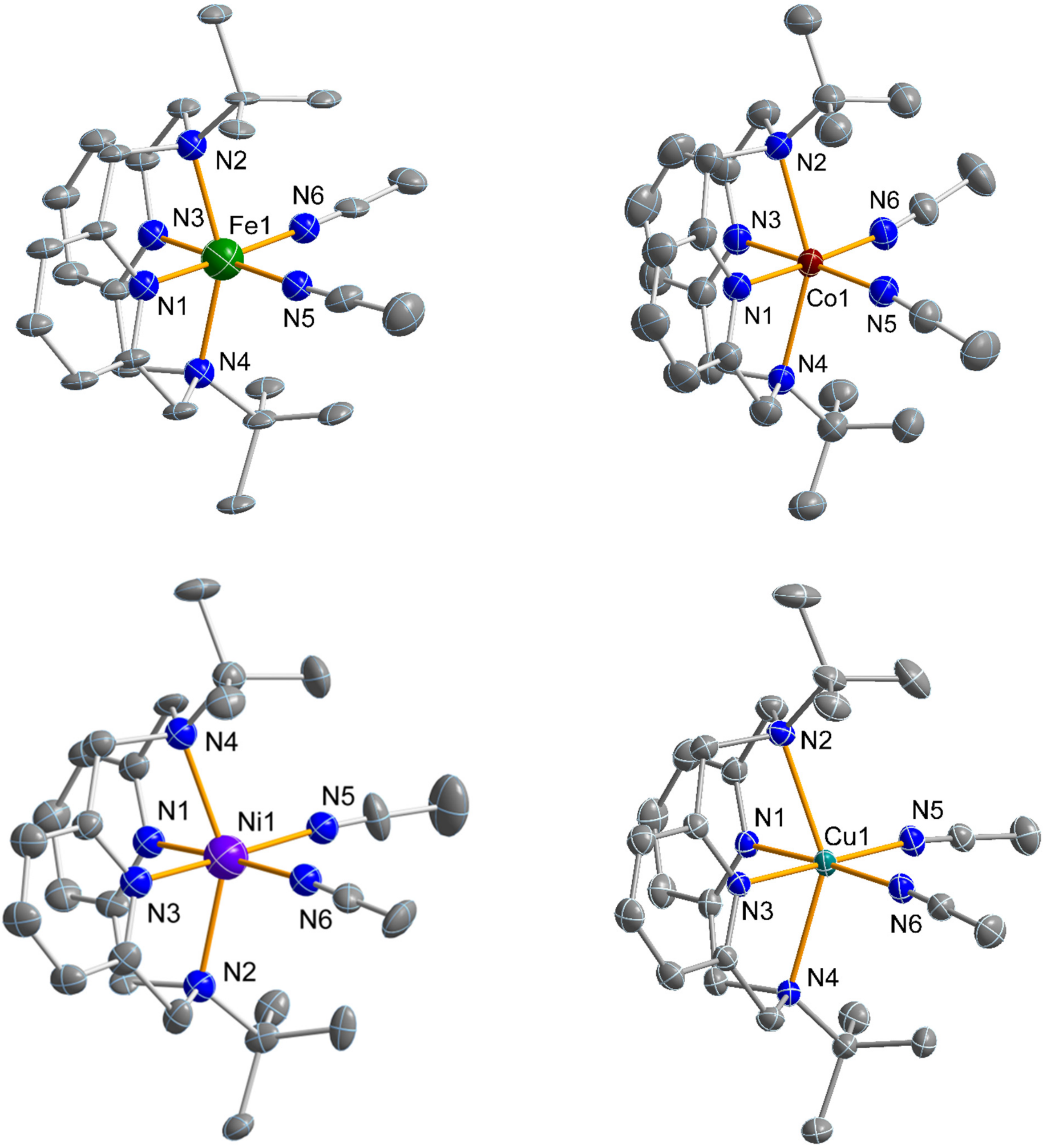

Figure S1. ORTEP diagrams of $[(^{tBu}N4)Fe(MeCN)_2](PF_6)_2$, $[(^{tBu}N4)Co(MeCN)_2](PF_6)_2$, $[(^{tBu}N4)Ni(MeCN)_2](PF_6)_2$, and $[(^{tBu}N4)Cu(MeCN)_2](PF_6)_2$ (cations only) drawn with 40% thermal ellipsoids.

Table S2. Selective bond lengths (Å) and bond angles (°) of metal compounds

	M–N_{pyr}	M–N_{amine}	M–N_{NCMe}	N_{pyr}–M–N_{pyr}	N_{amine}–M–N_{amine}
[a]$[(^{tBu}N4)Mn(MeCN)_2]^{2+}$	2.2359(18)	2.4150(18)	2.152(2)	75.10(7)	143.41(6)
	2.1647(18)	2.4554(19)	2.211(2)		
$[(^{tBu}N4)Fe(MeCN)_2]^{2+}$	1.877(10)	2.132(10)	1.932(11)	92.1(4)	154.3(4)
	1.875(10)	2.152(9)	1.946(11)		
$[(^{tBu}N4)Co(MeCN)_2]^{2+}$	1.909(3)	2.313(3)	1.917(3)	89.89(14)	152.04(12)
	1.903(3)	2.326(3)	1.935(4)		
$[(^{tBu}N4)Ni(MeCN)_2]^{2+}$	1.970(12)	2.265(12)	2.079(14)	88.9(5)	149.2(4)
	1.960(11)	2.246(12)	2.060(14)		
$[(^{tBu}N4)Cu(MeCN)_2]^{2+}$	1.9701(14)	2.4074(15)	2.0551(15)	87.60(6)	145.92(5)
	1.9964(14)	2.4075(15)	1.9805(16)		
[a]$[(^{tBu}N4)Cu(MeCN)]^{+}$	2.123(9)	2.436(17)	1.929(11)	80.4(4)	146.9(3)
	2.068(11)	2.458(17)			

[a]Literature values[2,3]

Mechanistic Studies

Table S3. Competitive aziridination reactions of 4-X-styrenes vs. styrene using PhI=NTs in the presence of $[(^{tBu}N4)Cu^{I}(MeCN)](PF_6)$ or $[(^{tBu}N4)Cu^{II}(MeCN)_2](PF_6)_2$[a].

**X**	σ_p	σ^+	σ_{mb}	$\sigma_{JJ}^{\bullet}$	**Cu^{I}** k_X/k_H	**Cu^{II}** k_X/k_H
t-Bu	-0.197	-0.26	-0.22	0.26	1.4474	1.4538
Me	-0.17	-0.31	-0.29	0.15	1.4519	1.4569
H	0	0	0	0	1	1
F	0.062	-0.07	-0.24	-0.02	1.0758	0.945
Cl	0.227	0.11	0.11	0.22	0.981	0.8609
CF_3	0.54	0.61	0.49	-0.01	0.7333	0.6513
NO_2	0.778	0.79	0.86	0.36	0.5903	0.5122

[a] Reaction conditions: catalyst, (5 mol%); 4-X-styrene, 1.0 mmol; styrene, 1.0 mmol; PhI=NTs, 0.125 mmol; MeCN, 0.30 mL; MS 5 Å, 20 mg; T = 25 °C; t = 3 h.

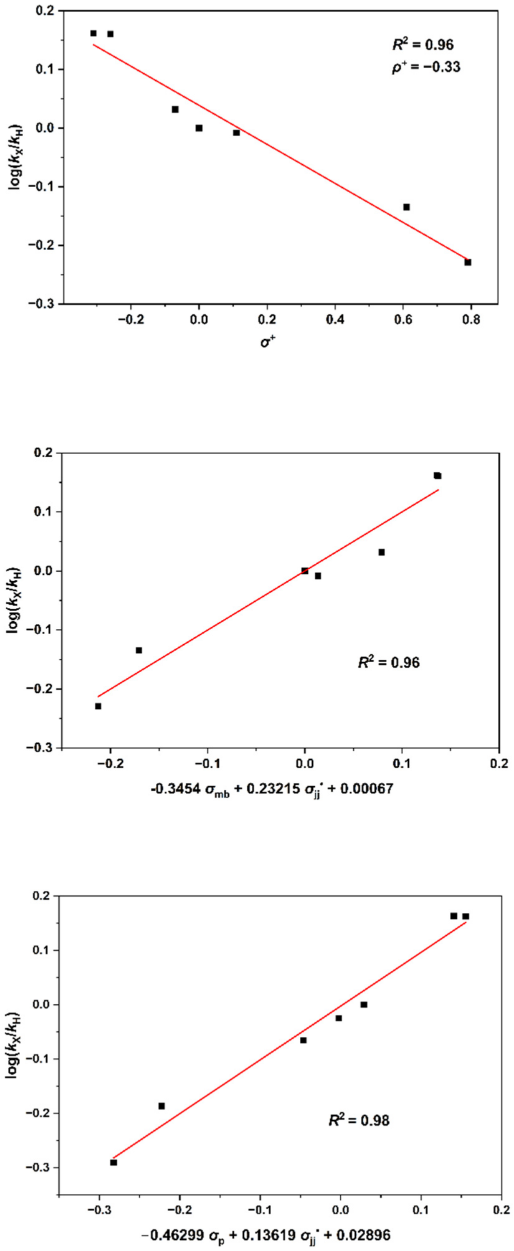

Figure S2. Linear free energy correlation of log(k_X/k_H) as a function of σ^+ for the competitive aziridination of 4-X-styrene vs. styrene catalyzed by [(^{tBu}N4)Cu^I(MeCN)](PF_6) (top); log(k_X/k_H) as a function of σ_{mb} and σ_{jj}• for the competitive aziridination of 4-X-styrene vs. styrene catalyzed by [(^{tBu}N4)Cu^I(MeCN)](PF_6) (middle); and log(k_X/k_H) as a function of σ_p and σ_{jj}• for the

competitive aziridination of 4-X-styrene vs. styrene catalyzed by [(^{tBu}N4)Cu^{II}(MeCN)$_2$](PF$_6$)$_2$ (bottom).

Representative Mechanistic NMR data

^{1}H NMR spectrum (CD$_3$CN) for the aziridination of styrene vs. 4-CF$_3$-styrene catalyzed by [(^{tBu}N4)Cu^{II}(CH$_3$CN)$_2$](PF$_6$)$_2$.

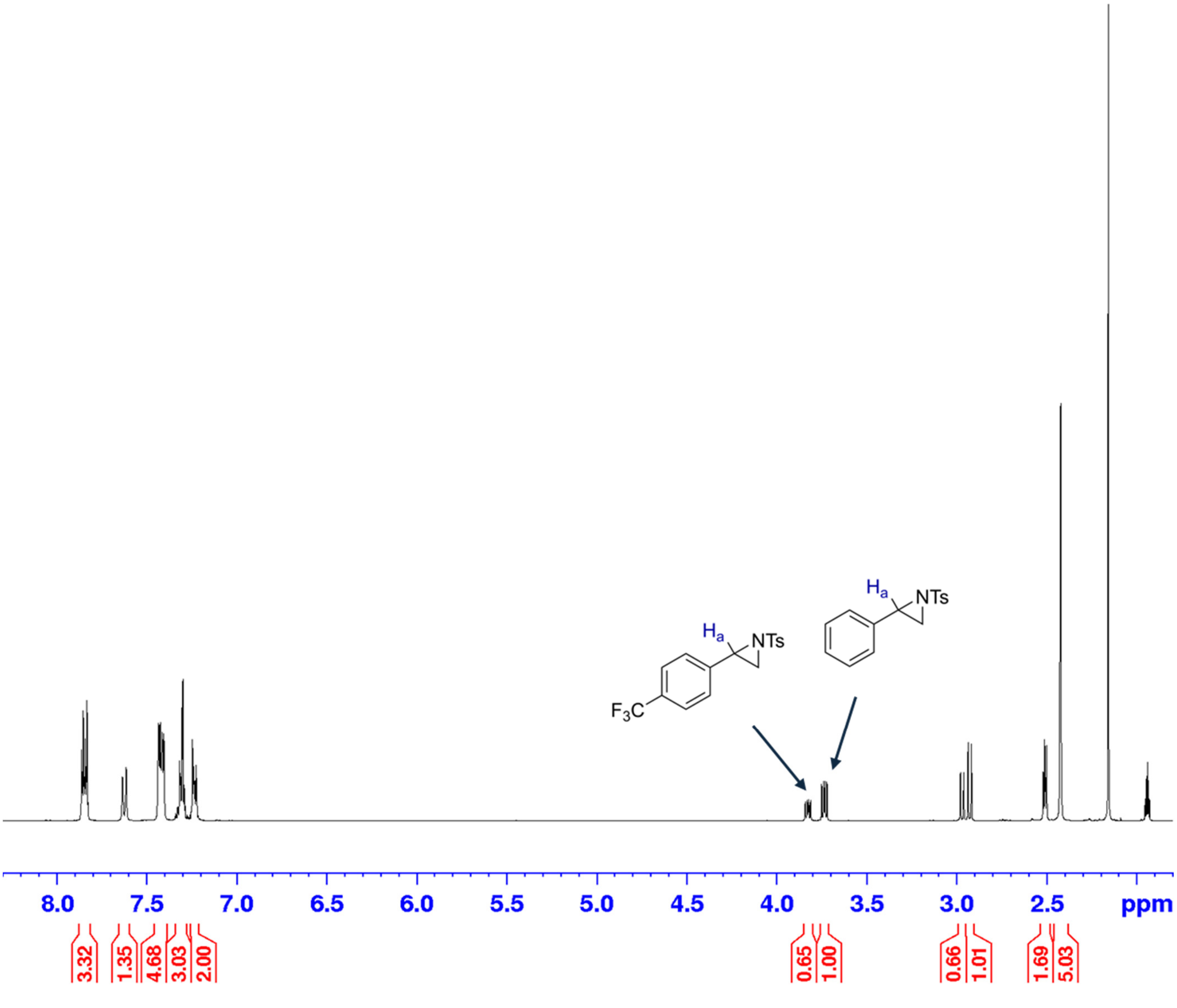

^1H NMR spectrum (CD_3CN) for the aziridination of styrene vs. *cis*-*b*-d_1-styrene catalyzed by $[(^{tBu}N4)Cu^I(CH_3CN)](PF_6)$.

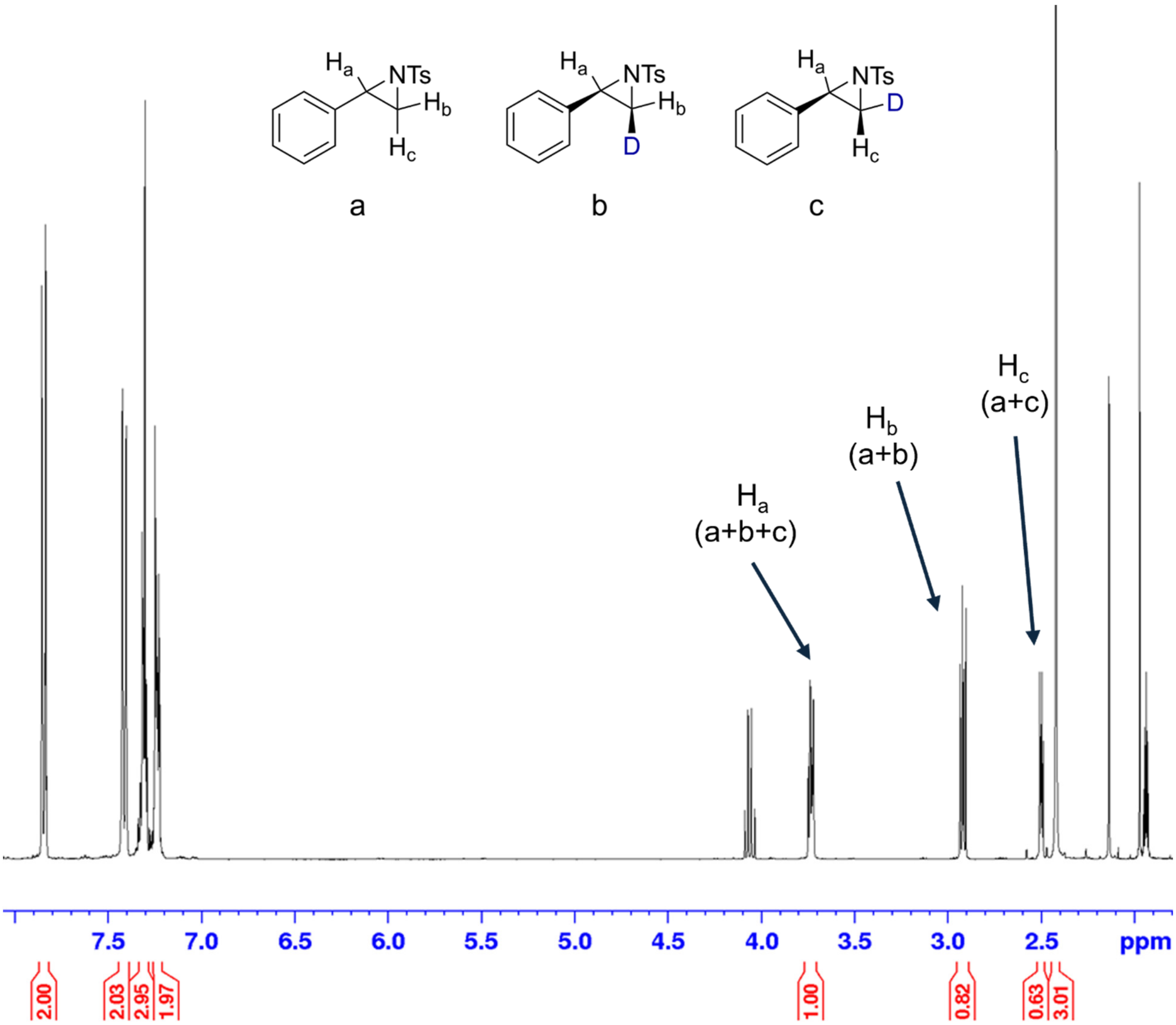

^{2}H NMR ($CHCl_3/CDCl_3$, (35/1, v/v)) for the aziridination of *cis*-*b*-d_1-styrene catalyzed by $[(^{tBu}N4)Cu^{II}(CH_3CN)_2](PF_6)_2$.

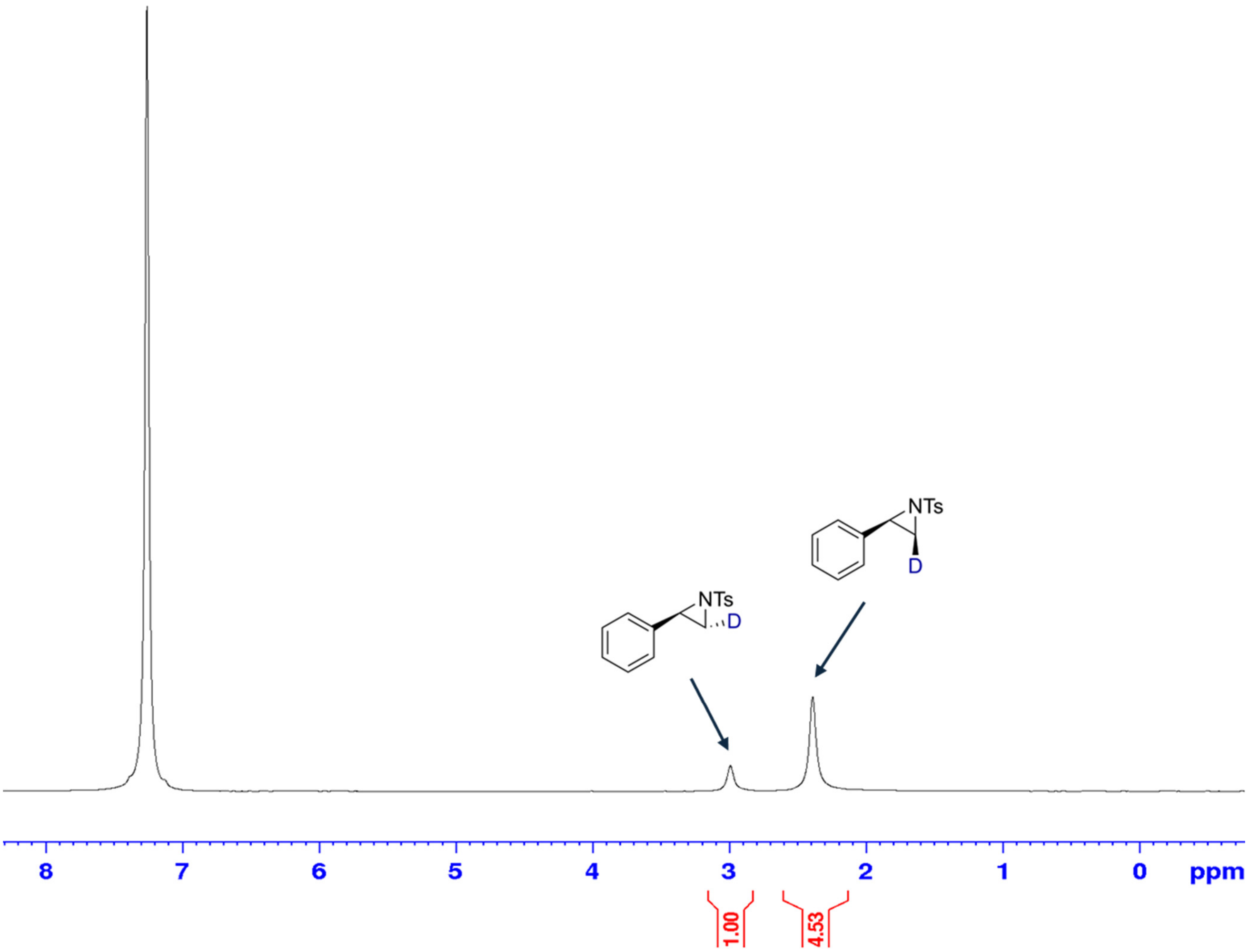

Computational Data

B3LYP/def2-svp/CPCM(acetonitrile)-optimized geometries of five- and six-coordinate copper nitrenes:

Broken-symmetry singlet 5-coordinate copper(I)-derived nitrene

Cu	3.95214015598992	4.34007067086742	4.42229086143699
N	4.86809694648856	4.46083089662274	2.62764886886978
N	2.31314835815802	4.97286772219967	3.29360886349258
N	3.23780048317077	2.20654350884246	3.39320879644046
C	4.74370992726336	3.43071117603357	1.76812132149941
C	1.70037195313694	4.05493811116290	2.53884883376258
C	3.25148496661063	0.87951486418771	4.18560067389055
N	4.39726154335554	6.72383606563770	4.30164076506794
C	5.28276220120395	5.66537085054960	2.18852683446881
C	2.24648204667719	6.28201541785957	3.02584977924322
C	4.98799668381559	7.43345840779099	5.53126027438813
C	5.50156494166929	8.84625934653380	5.17035639842510
H	5.80787061019527	9.36048190228158	6.09401332741330
H	6.37599233055729	8.82803880773296	4.50362644304707
H	4.71426907596462	9.45607043628881	4.69918548756919
C	3.93704068402835	7.58274005652589	6.64002910954063
H	4.44774838626417	7.89056207456259	7.56437320234086
H	3.18710731021712	8.35369551244978	6.41408858876813
H	3.43440737269258	6.63044382296742	6.84497622573125
C	6.14036356373081	6.57144531188397	6.07718794072761
H	6.61056076163579	7.09069340752634	6.92630226071693
H	5.75939118144953	5.60460002362999	6.43441359829923
H	6.93081164714421	6.38842718237701	5.33369110302011
C	1.97137397754103	0.69201093349516	5.01579370054879
H	1.10783378910150	0.42448624928369	4.38799956796328
H	2.13751559982854	-0.14562622877257	5.70958598536065
H	1.72040056728729	1.56862138526714	5.61876115422654
C	4.46127377526319	0.91738715026926	5.13230692393232
H	4.31743591624642	1.67742427084988	5.91294084144567
H	4.57651976653229	-0.05986772131318	5.62669187878882
H	5.40327080369906	1.12577309404635	4.60118806037752
C	3.34892272440283	-0.34621130373119	3.24475745917395
H	4.32597693274678	-0.44191771180373	2.75179107431231
H	3.19509182680537	-1.25860233184961	3.84031285620800
H	2.56624432923893	-0.32398770563680	2.46951686027749
C	5.33809501879539	6.80961425686327	3.16557574877176
H	6.36549742190934	6.83071725303337	3.55421702698894
H	5.20534786588034	7.75231283069208	2.60806611966425

C	3.05141095542711	7.21728781491438	3.91716092894138
H	3.11119710053557	8.20195373553991	3.42148602813626
H	2.46169195310039	7.35687667606496	4.83215946487783
C	4.22137200828503	2.12196516810637	2.29389963618940
H	3.82200779162632	1.54630637235407	1.44235026023901
H	5.09918485763275	1.56893147761912	2.65895840171621
C	1.89150683861267	2.59005641474792	2.90331795203589
H	1.17421675895106	2.39590043501203	3.70913114129333
H	1.58632414522515	1.97418940029123	2.03802297043839
C	5.68666738944137	5.85024212396470	0.86338489428548
H	6.02609098301865	6.83409918521946	0.53505579449108
C	1.43727979492914	6.75065930233130	1.98654872447208
H	1.37988712168934	7.81974698038435	1.77152118415259
C	5.62579573883434	4.77531671783643	-0.02181200436323
H	5.93305979759938	4.89703113314642	-1.06296322169405
C	0.72930879908623	5.81856184729689	1.22172691377301
H	0.09369859282170	6.15560768470062	0.39897555985666
C	5.12715215684522	3.55377355211313	0.43073783563549
H	5.01665410823278	2.70201310968569	-0.24292360141884
C	0.87160797763016	4.45312337719635	1.48327259963045
H	0.36490814409275	3.70613477488866	0.86900198248309
N	3.57085882718630	4.30217181611906	6.29111633928832
S	2.19423288027658	3.84585978282291	7.02451305638945
O	1.00642081499189	3.85141539563906	6.13880178935654
O	2.54955248793575	2.56508921868964	7.69381064751467
C	1.86941980557675	5.04260166349781	8.31490107525401
C	2.71261212896932	5.09293396364315	9.43426080833146
C	0.77680767033415	5.90572585037566	8.19919105747919
C	2.45145276849470	6.02329114529445	10.43796188335094
H	3.56114575164334	4.41034838859058	9.51882654430931
C	0.53079370285688	6.83332364226234	9.21598447419790
H	0.12424961833510	5.84660211771960	7.32655340496490
C	1.35962786070341	6.91076316974730	10.34726916531955
H	3.10647575906809	6.06352928386751	11.31307642240534
H	-0.32546319707642	7.50793233771301	9.12661549995316
C	1.08668434134927	7.90370025169809	11.44803693288874
H	0.75089191451108	7.38883954760658	12.36479163004523
H	1.99813270918762	8.46386248620357	11.71295353875712
H	0.30664628533993	8.62275889788607	11.15849361882058

Triplet 5-coordinate copper(I)-derived nitrene

Cu	3.84172021864747	4.66307321034558	4.32510564229049
N	4.85975984662593	4.63007712462367	2.54435200776018
N	2.23024027905292	4.93089659728806	3.10234612205430
N	3.49391175535790	2.35519173255711	3.61261758935756
C	4.86767121032332	3.49630838040595	1.81916920255649
C	1.74457565219357	3.83398316797832	2.51158208466170
C	3.74586908975413	1.21630656718243	4.61632771240654
N	4.10315026319550	7.02237181742002	3.88455386830908
C	5.18735471764952	5.79995986790693	1.96313115539605

C	2.06006552654436	6.16893628863157	2.62698031072496
C	4.58684634995041	7.92436819598372	5.03242282808434
C	5.02774061810124	9.31284104077462	4.51297427026981
H	5.24882301252275	9.96417420781735	5.37186374458325
H	5.93464075126586	9.27465580396892	3.89298099264245
H	4.22630586966046	9.79518590103216	3.93059911807804
C	3.49412456651344	8.15267149690528	6.08379362019671
H	3.94786432193488	8.66204633738263	6.94653960525647
H	2.67844809956239	8.79414862754112	5.71942765495735
H	3.06439267261078	7.21044889760153	6.43512349497712
C	5.76140613586345	7.20979037878318	5.72579942156348
H	6.16541687141977	7.84990927048520	6.52506090850356
H	5.42243018005622	6.26769458584822	6.18475964142294
H	6.59323823426646	6.98335535644299	5.04221162465847
C	2.61577549668996	1.09327719809750	5.64574015219301
H	1.68961626126440	0.69122605663168	5.20838478145119
H	2.93927065053488	0.38409819125752	6.42211407882530
H	2.38053750929026	2.04751636365443	6.12631265650585
C	5.04475435352635	1.54628244346668	5.37387955677526
H	4.92017504677358	2.46698163175253	5.96518534765506
H	5.28956010852594	0.72708363102125	6.06729427894798
H	5.91063079257813	1.67696714626830	4.70651881265222
C	3.86907904816350	-0.15243714106008	3.90465013468842
H	4.77859471227315	-0.24545536730623	3.29475589911160
H	3.90351292801573	-0.94873906270604	4.66381111936589
H	2.99802787018435	-0.34868404517940	3.25882931240592
C	5.08220238587165	7.05923271500090	2.78032422427824
H	6.08384891762281	7.23870345232912	3.19432571141152
H	4.88178044633185	7.90250545307628	2.09803946839194
C	2.72862128205284	7.30888738029538	3.39357777577439
H	2.70530972802585	8.21480854179303	2.76284779036688
H	2.08899689407795	7.48609114742116	4.26733741426097
C	4.41781721078059	2.21582781444524	2.47116586872619
H	3.99391836357719	1.56070375134200	1.69020769359124
H	5.33457849721385	1.71674496953551	2.81366913564719
C	2.08534108958977	2.49256927767012	3.15891909607335
H	1.43822717966637	2.41515148023695	4.04241477903361
H	1.80303330862326	1.68565761555173	2.46000481473108
C	5.64274405071228	5.85572618108811	0.64345432601644
H	5.90724004218054	6.81893244746354	0.20392100909590
C	1.25778691332719	6.36738926597248	1.49826893447524
H	1.10437200658144	7.37120364991746	1.09746342017896
C	5.72793551168175	4.67598082409956	-0.09457148452767
H	6.08042003061546	4.69226022372298	-1.12821929327130
C	0.68128006877981	5.24917257966869	0.88543531181614
H	0.05775244483422	5.37826926792932	-0.00268570864068
C	5.31295327651539	3.48282364372980	0.49413830496010
H	5.31218068322449	2.54513681280320	-0.06474304065549
C	0.93302189784905	3.96396074513477	1.37960740021141
H	0.52442429075023	3.07997210204566	0.88652260447764
N	3.11370012395798	4.64711044245082	6.08841597533324

S	1.53271229263730	4.89465752576929	6.47521953944185
O	0.96692968519731	6.11059724729918	5.85309287982638
O	0.80454708536387	3.62978298097991	6.21205902888370
C	1.55853171631301	5.15646927689415	8.24785571799501
C	2.06134973700014	4.15127656441843	9.08615641870175
C	1.06037814267790	6.34913686513396	8.77853035040149
C	2.06938357383280	4.35709791474389	10.46436299822220
H	2.45123347251897	3.22027029153294	8.66906499770750
C	1.07700264004018	6.53843381452278	10.16384313846720
H	0.66837983469971	7.12113750042240	8.11464267394828
C	1.57921595311324	5.55198451051327	11.02870873741984
H	2.46716096020181	3.57582109392606	11.11797983756968
H	0.69419855592053	7.47509910843178	10.57839282082807
C	1.57925419850390	5.75090900270248	12.52263974885002
H	0.72532325325531	5.22440833115170	12.98430991479589
H	2.49361575525128	5.34302304436246	12.98153785225606
H	1.49651406813478	6.81483748166504	12.78908921163782

Doublet 5-coordinate copper(II)-derived nitrene

Cu	3.63844859350175	4.68275524998774	4.22983574527376
N	5.09123878563732	4.71181792918639	2.91755118676557
N	2.19737082499349	4.82572312974737	2.82236092932300
N	3.66362916067976	2.44410616299736	3.43018984281705
C	5.71190971243555	3.56072755620846	2.59840843431630
C	1.92363718480263	3.75348276228645	2.07819792699353
C	3.10383387241706	1.31881208109216	4.30878484948452
N	3.63883424981456	6.95131954047180	3.78659107040001
C	5.40190344902313	5.87923419769683	2.32532668340731
C	1.60958491992498	6.01665350542619	2.66941387590330
C	4.22091807783036	7.88161469602842	4.85904625730847
C	4.03808832956957	9.35942786007906	4.44320127260348
H	4.53660549498366	9.99481291510872	5.18943458167663
H	4.48841613263569	9.58918003441113	3.46642630005565
H	2.97655790330807	9.64713155979789	4.41660978073666
C	3.52646268914084	7.67139526746656	6.20711658159958
H	3.95503930931735	8.37558923581571	6.93448880677392
H	2.44591519688191	7.85825581017015	6.16455937900062
H	3.70894109394512	6.65757683850741	6.59215034735972
C	5.71342259694452	7.55691675612495	5.03608137893475
H	6.10479630985667	8.15242914338495	5.87320730865973
H	5.86262134237908	6.49435629811376	5.28532281476104
H	6.32051364926652	7.80165114645969	4.15321392680964
C	1.57173320779533	1.41269975550879	4.34511358280971
H	1.10816986513361	1.11732338548922	3.39352944076961
H	1.19850624682858	0.72164307006286	5.11415426690612
H	1.22632329214582	2.41479124322583	4.62217343859793
C	3.66652813224417	1.43890023522220	5.73274673111090
H	3.38303627236237	2.39215100832930	6.19780442566283
H	3.24761854091516	0.62933173463471	6.34791188907405
H	4.76161475918889	1.34596636614665	5.77031711258005

C	3.50591480201997	-0.05842144952852	3.72843242271318
H	4.58460021569515	-0.25160172034071	3.81660124487565
H	2.98027809684733	-0.83678053975539	4.30050044647428
H	3.21104117207641	-0.16824308694861	2.67302326408536
C	4.42990749288492	7.01056223150260	2.51863632824960
H	4.94182856136300	7.97665896937145	2.42041269877925
H	3.73234172468062	6.96520433975510	1.66715136490375
C	2.18455035985868	7.14804541246493	3.50764162389616
H	2.01409813976844	8.10299890427183	2.98416888165042
H	1.64448244885678	7.19092699893763	4.46230493041607
C	5.13174498861688	2.31672800323105	3.20885210816870
H	5.35579049305950	1.45393180257917	2.56200727553557
H	5.61999909836813	2.13181341395279	4.17616219766232
C	2.91900974414104	2.60366895615537	2.14575401647152
H	2.42771905697903	1.66208184469584	1.86917290169371
H	3.65014278947274	2.79872977717905	1.34527535023141
C	6.49122537527876	5.95273867978091	1.45607279890378
H	6.75758838714534	6.90543110306297	0.99533838166192
C	0.56436739165249	6.15025266655690	1.75303631764404
H	0.07369015798873	7.11390110291640	1.60521062494093
C	7.20502168036125	4.78595499581612	1.17213597227280
H	8.06058797241296	4.82028893905739	0.49373433333201
C	0.17865355260290	5.01910406415129	1.02196991720825
H	-0.64708453790076	5.09171317889905	0.30996582492546
C	6.79347623550140	3.56670771155671	1.71808522037977
H	7.29009935197468	2.63124131770236	1.45633033566648
C	0.86989777947353	3.80981737961372	1.16101172675634
H	0.61583007343148	2.94077108511827	0.55084242061899
N	2.83100700972166	4.60644821282625	5.94397069235027
S	1.33485080594319	4.73858577927626	6.71369662582840
O	0.63527948995946	5.88668898618030	6.11774996583987
O	0.72791348411696	3.40256135231359	6.64434887196430
C	1.77886347345348	5.08736983979712	8.38748871456571
C	2.26398419138271	4.04103366449105	9.19409416923345
C	1.60063164900696	6.38155033195650	8.89681340396402
C	2.58085408428780	4.31186431573848	10.51980415689462
H	2.38134705576473	3.03219429692846	8.79420763631043
C	1.91472139062916	6.62518724353983	10.23203531166678
H	1.20424969169718	7.17675674661683	8.26470805844223
C	2.41202924142533	5.60309994846103	11.06390596795523
H	2.96000092436597	3.50609968566950	11.15316359998534
H	1.76619244349838	7.62809825598008	10.63930400054520
C	2.73759921441790	5.86575278144806	12.50757700414735
H	1.99701855005209	5.37232827436578	13.16091861624589
H	3.72211585271615	5.44818996795808	12.77243523767339
H	2.73306823704445	6.94046399950624	12.73720301779221

Quartet 5-coordinate copper(II)-derived nitrene

Cu	3.00658149624776	4.75910375138071	4.18722899123539
N	4.86536427930303	4.73559157331894	3.32230686304042

N	2.36725475692380	4.85157882121621	2.24970676658509
N	3.16836028864165	2.46850996325549	3.58774519256402
C	5.31756486413531	3.56242254580515	2.84873097346529
C	2.37363722406886	3.71562085586890	1.53501319163334
C	2.65261885588058	1.42027086623506	4.59133741996700
N	3.29855043924440	7.07439731471579	3.76380163424062
C	5.36383639524878	5.89986344198131	2.87892631357269
C	2.38788392158363	6.05395136065665	1.65022502114243
C	2.93692626776771	8.07680426175468	4.87584715240778
C	3.33710453414219	9.51201296036533	4.45240486524457
H	3.00912756248340	10.20338137270819	5.24289012166693
H	4.42374997476554	9.63231021888716	4.33814688359427
H	2.84275444438171	9.82098451711720	3.51926215887964
C	1.42289471976933	8.05200228054133	5.13922850860599
H	1.22299582907501	8.57663707841203	6.08416955915338
H	0.85043240733766	8.57298430893196	4.35945730913468
H	1.03393700682999	7.03186003871211	5.24351151922606
C	3.69137486893133	7.70922954252542	6.16281115802132
H	3.34217474120456	8.36257048265050	6.97428282327674
H	3.50454981291134	6.66792685442889	6.45729127683524
H	4.77866289177365	7.84868275947235	6.08636867189903
C	1.13088335975704	1.56565617641242	4.76354559803919
H	0.57149421814280	1.13616940602846	3.92102011813949
H	0.82392402570473	1.01882286215187	5.66500314651353
H	0.82495700988434	2.61053743229126	4.89732671093787
C	3.36062069133876	1.61051738151262	5.94253901494691
H	3.31090336283351	2.65397585678397	6.28014993355082
H	2.86485595025605	0.98275533897274	6.69571078701415
H	4.41721405218135	1.31051642528913	5.91706316803696
C	2.95359552518744	-0.00754124397245	4.07426031821374
H	4.03159265135543	-0.21505146057031	4.01428999771986
H	2.51852404951602	-0.72463713431578	4.78607966873003
H	2.49672688304917	-0.19902051220350	3.09190022075249
C	4.74826754500002	7.16189350974963	3.42056389575182
H	5.30515539964322	7.42103582673060	4.32970670375778
H	4.91133488923061	7.98273634325487	2.70518134405527
C	2.43881481674900	7.25095755274928	2.55826198970881
H	2.76335701667414	8.13552961855919	1.98779952593450
H	1.41188253160580	7.44157421728258	2.89164315769218
C	4.63465456812048	2.32245980232366	3.35721318508252
H	4.82071769341416	1.49232279745333	2.65846157242062
H	5.11037655837757	2.05553741436882	4.30929091874380
C	2.40371170742529	2.42453033341184	2.30736406472890
H	1.36205522815947	2.17072154095759	2.53533273253371
H	2.79363557216954	1.61885230998663	1.66577408129783
C	6.43591481076506	5.92671085804968	1.98332715471159
H	6.83517577336236	6.88115168223078	1.63621149773043
C	2.32306782768419	6.16069299014437	0.26009552668139
H	2.33628303843535	7.14275537546630	-0.21553375823667
C	6.96284673903027	4.71545427893968	1.53251502583919
H	7.79861309546186	4.70766632401779	0.82906639538445

C	2.26448987891798	4.98927400482488	-0.49977571006007
H	2.21852005517292	5.04391448953207	-1.59036606198997
C	6.38736737457579	3.51415049868948	1.95328997620771
H	6.74752349111027	2.55370235330207	1.58115781727630
C	2.30738872717619	3.74898686909615	0.13968337588832
H	2.30710125590338	2.81826638030170	-0.42988562883324
N	2.21135794720133	4.71164780078736	5.90865076888527
S	1.23521459095678	4.65236013733748	7.29453788115452
O	0.31997518160327	5.79263319760060	7.18123911060358
O	0.70224970661600	3.28728826675638	7.33783617041257
C	2.34307560874061	4.89830987770835	8.64583885783741
C	3.05230773976301	3.79972281025299	9.16297488200370
C	2.44330825619914	6.17288958086538	9.22525493653252
C	3.87445958444198	3.99475337666921	10.26764658310027
H	2.94864312124829	2.80840148524753	8.72091418063827
C	3.26783119903878	6.34090519198283	10.33408899978103
H	1.86893192815458	7.01046805818798	8.82789664675144
C	3.99544123172454	5.26174596918445	10.87486676975078
H	4.42578712031519	3.14483934469554	10.67697289106372
H	3.34269110736231	7.32801748252963	10.79686515391745
C	4.85297462219344	5.44643523485674	12.09542126872901
H	4.27852193229377	5.17900693926490	13.00057297799041
H	5.73847060088884	4.79384483679393	12.06878552003443
H	5.17762215523478	6.49073157853330	12.20820071051878

Broken-symmetry singlet 6-coordinate copper(I)-derived nitrene

Cu	4.10719775410595	4.75290713702600	4.52926331397845
N	5.00360769824165	4.62768502504702	2.61031535319474
N	2.49751212568341	4.95791870435024	3.30960693503612
N	3.70632330260489	2.33684445080728	3.86750686818070
C	4.96888940736255	3.44562427588316	1.96785690005973
C	1.94092944350456	3.84422345964985	2.80808954676125
C	3.83160028069384	1.19031306350210	4.87593562987934
N	4.33928976968997	7.18351573948256	3.82896110473056
C	5.25484004745553	5.76368499276473	1.93593202857441
C	2.23340744037958	6.17740953697756	2.81303745692197
C	4.78977020871274	8.25068373079894	4.82969421601043
C	4.54236567591716	9.67743609392706	4.29093737098024
H	4.97321917184135	10.41245015457944	4.98845199305838
H	5.01979003319844	9.83718143495453	3.31067674239132
H	3.47036539059358	9.90693271761156	4.19494956593796
C	4.03513061028082	8.06856130362273	6.15681496563716
H	4.36980253595153	8.84001594529420	6.86724853603362
H	2.94657930175129	8.16321337539546	6.05186710514301
H	4.24220165569187	7.08090196516717	6.59507297119283
C	6.29203589085858	8.10121284174786	5.14267309224978
H	6.53048019719660	8.71761787887214	6.02207593392632
H	6.54668578915298	7.06027299494087	5.38242888593490
H	6.94495585387700	8.44736419634665	4.32816486614116
C	3.11084157133105	1.56265965841550	6.18109840246494

H	2.03931789196470	1.75417178340169	6.04461667139190
H	3.21370601664767	0.73141746609508	6.89612174698859
H	3.55411875720967	2.46410017099108	6.62978542240123
C	5.31004774013337	0.93699232486427	5.23356776473192
H	5.82865167589332	1.87459398988116	5.47582608453815
H	5.34973801612982	0.29222343805481	6.12425651691094
H	5.86686583645237	0.41612770561145	4.44062524437161
C	3.23486818702918	-0.12171384454993	4.31627371314828
H	3.68469360621966	-0.39529943577149	3.34849788844210
H	3.43717084038598	-0.94562321492644	5.01815971347457
H	2.14371890885937	-0.06043641883350	4.18717181837824
C	5.23570336204106	7.09119201644297	2.66764759033850
H	6.26805184010524	7.25613961312780	3.00101027287936
H	5.02711975460970	7.88005172760950	1.92123006783357
C	2.93846887696702	7.38518831745703	3.40556760062954
H	2.84823245849437	8.21031358034443	2.67649259650854
H	2.34860081717100	7.66306681350737	4.28950449712693
C	4.64449620770541	2.18737123598583	2.74500576674810
H	4.30813578256124	1.42148268674014	2.02196062672809
H	5.60551655517891	1.83345718139516	3.13820041577170
C	2.31595622299912	2.49372339134619	3.39403648145755
H	1.64479580589436	2.35562556219923	4.25279158098006
H	2.05241843281110	1.72652248527097	2.64484803660688
C	5.57821233985282	5.73733326162024	0.57418062158964
H	5.78627213427467	6.67201898500075	0.05024022665685
C	1.28623426104447	6.32902688869690	1.79473624301119
H	1.07288344991440	7.32148829875771	1.39342711752233
C	5.60890993054524	4.51458532158039	-0.09352782313734
H	5.85555905214524	4.46928280965973	-1.15694917790735
C	0.64626895338039	5.19511775939453	1.29124013554319
H	-0.09261289416962	5.29078879496313	0.49164154621451
C	5.27988393370528	3.35453550454394	0.60693272515283
H	5.24734053226142	2.38387010604753	0.10802667699155
C	0.98694962220487	3.93626557465897	1.78818892267140
H	0.53501574631569	3.02875251974807	1.38382339496057
N	3.13955406446511	4.86631048816692	6.28945294652244
S	1.53766492434761	5.06005275517338	6.46211293512028
O	1.14342854026818	6.40769142435278	5.97506367651449
O	0.79022572782486	3.90420434392992	5.90504530472603
C	1.22423500440133	5.05876706539070	8.23425833915050
C	0.71194107472556	3.91271382357018	8.85127076803561
C	1.50855954428172	6.20479127885682	8.98578732067297
C	0.49149781673123	3.91793829198464	10.23065442782703
H	0.47960314079805	3.02886493004632	8.25483668397234
C	1.28426591530739	6.19307839274441	10.36414672692877
H	1.89682093008918	7.10311348405246	8.50148379451844
C	0.77254260638809	5.05352981317299	11.01136040791901
H	0.08906831168050	3.02032661076254	10.70923374700095
H	1.51042915850433	7.08964955541011	10.94851517962735
C	0.50574439216636	5.05281558405722	12.49559806517871
H	-0.57136826590619	5.18852885356040	12.69754937114947

H	0.79994868401399	4.09606506241823	12.95518013787616
H	1.04438067185960	5.86562496763645	13.00443780311249
N	5.91787622647945	4.51471176160860	5.51717929950688
C	6.91570750970710	4.37730353291543	6.08320561120480
C	8.16791553977934	4.20406989884723	6.79506539639687
H	7.99023662116693	4.27975348203798	7.87879066634005
H	8.58893019513500	3.21402136663159	6.56173512512617
H	8.88018277876856	4.98353749858985	6.48412139549545

Triplet 6-coordinate copper(I)-derived nitrene

Cu	4.10411814475194	4.75326472815769	4.53656404705509
N	5.00382381209503	4.62728310423911	2.60944010098461
N	2.49325223484696	4.95916224355498	3.31645142842092
N	3.70592810191149	2.33744456637768	3.86883587791821
C	4.96304534296287	3.44629443507271	1.96568958232008
C	1.94157339600031	3.84536022936916	2.80933538516600
C	3.83130724810711	1.19080693079680	4.87664643665068
N	4.34056076515557	7.18320041922418	3.82936649610611
C	5.24933843344227	5.76329866387553	1.93325491494849
C	2.23451517071177	6.17756618645134	2.81409047234170
C	4.79148610586933	8.25035136255017	4.82954228198034
C	4.54452595093090	9.67720645434751	4.29066373544625
H	4.97626811583975	10.41221769503704	4.98763834881000
H	5.02133272798795	9.83631147012115	3.31001784028882
H	3.47258201309260	9.90729021940632	4.19526324704796
C	4.03681152941201	8.06909121101519	6.15684192554630
H	4.36927472946970	8.84262182622304	6.86606021368092
H	2.94809699550709	8.16132092422068	6.05107611956003
H	4.24655802039741	7.08295933512749	6.59716667406216
C	6.29369129601011	8.10062954326158	5.14260897137724
H	6.53230752234757	8.71689261254201	6.02210446777374
H	6.54797873329164	7.05953023327527	5.38220671821233
H	6.94649149201310	8.44668915729363	4.32797371668738
C	3.11097439906725	1.56264311666043	6.18229602604822
H	2.03978474019317	1.75614688771050	6.04561839643322
H	3.21163837809994	0.73003699011266	6.89603777920021
H	3.55655187334754	2.46196963029519	6.63280525439541
C	5.30990104548168	0.93739653208526	5.23371880656033
H	5.82884581099809	1.87522640898310	5.47437522475497
H	5.35014144120592	0.29338767447716	6.12495798065543
H	5.86591195730570	0.41588888696769	4.44065711673259
C	3.23406274452873	-0.12111011860812	4.31710991596622
H	3.68283675057924	-0.39415198321994	3.34869102548086
H	3.43721367506209	-0.94540222523954	5.01830125870763
H	2.14275831352808	-0.05985243111359	4.18919002660662
C	5.23762488071657	7.08906659785619	2.66827539756307
H	6.27045695309675	7.24686923414501	3.00354442249819
H	5.03402441270553	7.88150169295133	1.92420588731869
C	2.94014364705691	7.38590752840688	3.40527627986482
H	2.85161729214241	8.20932576005792	2.67398437222855

H	2.35037478247975	7.66738804265416	4.28802684040332
C	4.64466279535951	2.18855876173033	2.74617310174735
H	4.31101299968565	1.41906886432694	2.02569261709531
H	5.60704113124567	1.84004440742437	3.14072436319271
C	2.31566288411404	2.49444045742443	3.39532107875019
H	1.64424488427165	2.35545116155874	4.25363621583058
H	2.05240652126085	1.72769563057182	2.64551942979560
C	5.56042971475260	5.73866849323129	0.56851471819060
H	5.76394979337927	6.67366915723597	0.04334784386243
C	1.29492418385429	6.32856492148386	1.78865089120926
H	1.08590458481162	7.32057878559589	1.38392539670429
C	5.58505116385172	4.51639821075702	-0.10047064567639
H	5.82238221036275	4.47193582532289	-1.16609387014860
C	0.65764232341187	5.19465261111683	1.28196209863586
H	-0.07546628932425	5.28971484948211	0.47699101223812
C	5.26182334494185	3.35575152144825	0.60185586177449
H	5.22469959763574	2.38565520212527	0.10210140249735
C	0.99553507302818	3.93628432015807	1.78201402041171
H	0.54795487833317	3.02820915439657	1.37406229763398
N	3.13753364540796	4.86678109333902	6.28557021456022
S	1.52729004932966	5.06284214974749	6.45318904431935
O	1.13787141761781	6.41082955613574	5.96715524032089
O	0.78334841483297	3.90633627274784	5.89579545772231
C	1.22488100966646	5.05899246137003	8.22613635365795
C	0.72099143693484	3.90986135891034	8.84437030201852
C	1.50658929462118	6.20663661285386	8.97612842500744
C	0.50628859830057	3.91382814069551	10.22451227615925
H	0.49055703513064	3.02501258043333	8.24868186197138
C	1.28830484640367	6.19323962566898	10.35534145327215
H	1.88807414570353	7.10694875604029	8.49024640947586
C	0.78514823390724	5.05077424049582	11.00420881871995
H	0.11032978982435	3.01421962760980	10.70461595268674
H	1.51256360789013	7.09068953373607	10.93902517796043
C	0.52484140998065	5.04832603174164	12.48946353896821
H	-0.55174446234384	5.18152856084576	12.69581213594244
H	0.82302057288493	4.09183557762627	12.94693705590122
H	1.06372141680081	5.86203881001245	12.99656596531067
N	5.91031848990258	4.51596788867365	5.53874373790057
C	6.90475900458061	4.37887968521060	6.11092420729775
C	8.15284125566580	4.20568029940452	6.83030550501645
H	7.96830725218830	4.27736561398188	7.91315277820105
H	8.57747381891346	3.21728972214607	6.59650115166382
H	8.86561986512840	4.98757103245831	6.52668173039515

Doublet 6-coordinate copper(I)-derived nitrene

Cu	4.09642745420310	4.75478887000549	4.44168829981943
N	5.01341011468458	4.61858513443846	2.62036585102741
N	2.49804904972747	4.96704972994388	3.29044933491608
N	3.67978340656243	2.36462296404649	3.89371656163035
C	4.99571364344749	3.42416416464722	2.00108349571635

C	1.92796245208435	3.85182303220727	2.79889876077685
C	3.77106699435214	1.23646782394051	4.93534634246399
N	4.34067303602600	7.16007955446473	3.84279510892907
C	5.29172699580146	5.75389919608787	1.95702856983410
C	2.23486142219480	6.19497936095223	2.80457870933487
C	4.77386888053086	8.21351802417627	4.87508793712439
C	4.50476137366779	9.64451723979065	4.36063525288559
H	4.93214889281256	10.37024387650503	5.06920324938135
H	4.97746720512464	9.82402930064028	3.38179783885123
H	3.43056386477936	9.86494306096917	4.27451273861869
C	4.00689187368530	7.98593597253099	6.18697825302554
H	4.29942305662969	8.75934423055559	6.91287696764708
H	2.91708927895428	8.04822859833848	6.06441399405046
H	4.25575895722707	7.00641033990072	6.62371538486234
C	6.27616885725660	8.08759528655157	5.19165215610741
H	6.49338664505868	8.68555425580687	6.08880776918009
H	6.56127964159092	7.04927073805571	5.40571868340916
H	6.92211108870980	8.47437137043093	4.39030117305073
C	3.01086590696394	1.64579881338423	6.20536494731735
H	1.95133226761277	1.86451761110149	6.01921750800784
H	3.05067282718236	0.81834996159031	6.93025145055290
H	3.47411578762569	2.52697794263398	6.67495772379426
C	5.23575841328967	0.97725299861143	5.33726793632444
H	5.76309088225783	1.91049618381003	5.57549421353662
H	5.24206820867333	0.35128529438393	6.24164800305597
H	5.80581868319157	0.43289868457830	4.57022597679908
C	3.17788168639130	-0.07914510201571	4.38164813044688
H	3.65045962644613	-0.37112044162019	3.43073657306630
H	3.35998312661419	-0.89021694816198	5.10312712581710
H	2.09080549704489	-0.01545434960400	4.22593725495145
C	5.27414570861889	7.07086161739749	2.70392977703343
H	6.29815964886233	7.20903868344817	3.07248946301414
H	5.09874783348862	7.87572878517431	1.96848493019080
C	2.96086483808328	7.39814143867152	3.37372234095135
H	2.92456966047098	8.19430435527521	2.61048456975028
H	2.35229240562911	7.74272815592155	4.22124093733494
C	4.66302066353401	2.18795999220214	2.80583111354487
H	4.35529289348750	1.39409440863678	2.10358996122699
H	5.61362921072485	1.86189690067512	3.24569624751025
C	2.30967091110016	2.49511036587469	3.35920655067972
H	1.59368231856647	2.29713109515152	4.16896115506829
H	2.10813519273052	1.74860405463924	2.57193891888324
C	5.65061541576701	5.71170909717534	0.60606302608064
H	5.88114662849428	6.63945440957659	0.07985520179885
C	1.26768217879894	6.34868295049942	1.80853233963541
H	1.05349071048475	7.34368958659655	1.41487397581728
C	5.68839466004862	4.48060682274484	-0.04764709780613
H	5.95898362100355	4.42594796646865	-1.10452311199505
C	0.61053108298853	5.21945768016980	1.31782048318230
H	-0.14611456737315	5.32173323013125	0.53630389317784
C	5.34306746491376	3.32311338989619	0.65148814489684

H	5.32626582782500	2.34855737860841	0.16011800616199
C	0.95460372620667	3.95713045187070	1.80166271709657
H	0.49073667859855	3.05239795886039	1.40527961625105
N	2.89791799793026	4.88106122773570	6.08951401077685
S	1.23058901250318	5.13184634730189	6.29236674341524
O	0.94068546440277	6.48927524040988	5.80843173624043
O	0.51989218695290	3.98409383320614	5.71238098265652
C	1.15847199419940	5.07822379179627	8.05384080427047
C	0.87546199578323	3.85860177334548	8.69598618867120
C	1.40200215207398	6.25527396390777	8.78500375282539
C	0.83072783363195	3.83133319699793	10.08451826417386
H	0.67645976247312	2.95684145079535	8.11470831133729
C	1.35442671129385	6.20041009106736	10.17358656963257
H	1.61194675219945	7.19612954342832	8.27390027421531
C	1.06982526028888	4.99484917349197	10.84815312295292
H	0.60113484782954	2.89140177970244	10.59239969936079
H	1.53974382536537	7.11038594235344	10.74943779288797
C	0.99119867592241	4.95036585648821	12.34682653756631
H	-0.06487804564859	5.00250511590892	12.66707386411083
H	1.39905299683959	4.00738962335297	12.74169248801752
H	1.52126763080250	5.79572856634119	12.80821847234573
N	5.76620976336284	4.54580680678549	5.55438913837560
C	6.71561631097513	4.42257647009189	6.20050182937484
C	7.90546816134660	4.26615985791388	7.01066216854295
H	7.63196482515396	4.31338107748442	8.07596980753189
H	8.37031091070363	3.29226557395682	6.79218280242514
H	8.61796408615474	5.07179949079088	6.77522584449240

Quartet 6-coordinate copper(I)-derived nitrene

Cu	4.03462685209754	4.72774812593260	4.47748859956933
N	4.96599299413275	4.62180342417532	2.60061359547599
N	2.43983914197294	4.95013903935397	3.30007955023580
N	3.63402315785817	2.37028413640636	3.86443507961096
C	4.94874565291129	3.45157950831369	1.95106670588264
C	1.89289926068586	3.83990245022086	2.77197008651557
C	3.80405450084419	1.21681530404185	4.87794915490782
N	4.26409497951421	7.11060418417553	3.91298626561683
C	5.25964093101437	5.76854108967905	1.97505158296544
C	2.20244944552194	6.17475861723207	2.79502929977296
C	4.72536730759114	8.14808760840903	4.95975682133524
C	4.91330687732926	9.52734291625507	4.28322494045685
H	5.13625869861879	10.26828442075244	5.06532675933228
H	5.75195030093720	9.53619586557208	3.57181021969300
H	4.00293077629972	9.86205705739239	3.76334471988044
C	3.69463608995009	8.28705871711631	6.08939828738975
H	4.14485352994179	8.88855715532918	6.89218761803993
H	2.77325274914206	8.79959060100469	5.78197206131494
H	3.43349685917679	7.30765077121934	6.51146178252094
C	6.04939343128950	7.71203739356336	5.60873782865858
H	6.42177483206735	8.54562026486727	6.22166009122738

H	5.89441636807240	6.85290397233021	6.27219023122204
H	6.84123759864051	7.46656975717953	4.88775200253550
C	2.78669548838482	1.33272434988910	6.02257433193125
H	1.75361524172917	1.11719674192586	5.71677245689899
H	3.05481232862739	0.59361059867811	6.79116698772351
H	2.82256944314515	2.32574990397619	6.49035103903733
C	5.20149699774969	1.25295272293246	5.51871177210144
H	5.30442419773504	2.11860603771609	6.18392856454817
H	5.32078135052121	0.34712610022119	6.13080289483927
H	6.02748835312271	1.25969306630995	4.79398186837221
C	3.60072227632733	-0.14143733011085	4.16420545415981
H	4.39245817054316	-0.35574562202122	3.43201020654660
H	3.62600105315157	-0.93929782064130	4.92177947366418
H	2.62503112817599	-0.20067824991228	3.65878856078901
C	5.20613759370199	7.05864909137183	2.76508603178753
H	6.22156191468207	7.24127151516333	3.14004404572884
H	4.98232680274340	7.87950659482187	2.06450135265892
C	2.86905538920492	7.34723731144783	3.46673877542772
H	2.81790515572050	8.22241979427484	2.79848660546302
H	2.25656107711095	7.56765830425874	4.35026899452453
C	4.55729013658255	2.20332568179727	2.71282056189441
H	4.12925679057944	1.48668134277544	1.99240740281782
H	5.48947253457623	1.75241867832874	3.07682851433564
C	2.22601742649822	2.52136351122592	3.42127834440274
H	1.57615344903126	2.45566950805976	4.30289866353961
H	1.94424314031918	1.70474492653174	2.73748939073362
C	5.64878854812888	5.76831589087976	0.63228925366042
H	5.89217115342994	6.70714150818457	0.13197177809469
C	1.31079432304743	6.33357503004044	1.73233450749242
H	1.11883636850242	7.32884803507753	1.32884892144932
C	5.69792636029646	4.55147872615218	-0.05053973881226
H	5.99491546881244	4.52294332715049	-1.10125499950451
C	0.69514479139816	5.20251689178208	1.19240893622225
H	0.00128421965156	5.30397293525251	0.35475501925206
C	5.32653914916690	3.37707784973629	0.60688179965614
H	5.31201563170639	2.41786718777451	0.08588585973230
C	0.99491478031138	3.93946460548787	1.70713076859237
H	0.55239121763511	3.03638563787899	1.28452321804045
N	2.96502166582326	4.83390059638411	6.25257378961309
S	1.31788178641961	5.06093559502928	6.45424749815930
O	0.95204983796578	6.37939000003248	5.90389478608111
O	0.62608983063501	3.86161341473781	5.94528860078562
C	1.17374546333051	5.11559778073795	8.22145135375778
C	0.96010549056285	3.92469467067097	8.93430072210085
C	1.30764060785679	6.34668877115227	8.88259810482746
C	0.88357401172057	3.97768640988797	10.32295043908474
H	0.84231112624668	2.97667010582944	8.40642862493187
C	1.23133701656091	6.37517025711242	10.27263477275474
H	1.45901353398189	7.26711928921040	8.31638327144039
C	1.02076175185881	5.19745041693241	11.01721277737635
H	0.71094296083318	3.05509020177257	10.88314107053211

H	1.33624317900310	7.33087808869324	10.79281305506271
C	0.91377024649434	5.24137711200426	12.51621640315404
H	-0.14768462909088	5.28560723145181	12.81886762405608
H	1.34299156961627	4.33839219863422	12.97627627789112
H	1.41293630954671	6.12841307551637	12.93263934128415
N	5.80076755294086	4.47349406947184	5.41205723041812
C	6.85387721780113	4.30296618727313	5.85316650122516
C	8.16979422159138	4.07885892296326	6.41430169454023
H	8.15101518399682	4.29964556553325	7.49249364903263
H	8.45765775507085	3.02761312682696	6.25864488166887
H	8.89963744557199	4.73342949120069	5.91467027025843

Accession Codes: CCDC 2456020-2456023 contain the supplementary crystallographic data for this paper. This data can be obtained free of charge via www.ccdc.cam.ac.uk/data_request/cif, or by emailing data_request@ccdc.cam.ac.uk, or by contacting The Cambridge Crystallographic Data Centre, 12 Union Road, Cambridge CB2 1EZ, UK; fax: +44-1223-336033.

References

(1) Che, C.-M.; Li, Z.-Y.; Wong, K.-Y.; Poon, C.-K.; Mak, T. C. W.; Peng, S.-M. *Polyhedron* **1994**, *13*, 771–776.

(2) Lee, W.-T.; Muñoz, S. B., III; Dickie, D. A.; Smith J. M. *Angew. Chem. Int. Ed.* **2014**, *53*, 9856–9859.

(3) Filonenko, G. A.; Fayzullin, R. R.; Khusnutdinova, J. R. *J. Mater. Chem. C.* **2017**, *5*, 1638–1645

(4) Bruker's APEX3, SAINT and SHELXTL. **2017**, Bruker AXS Inc., Madison, Wisconsin, USA.

(5) Bruker's SMART **2002**, Bruker AXS Inc., Madison, Wisconsin, USA.

(6) Bruker's SAINT, SADABS, SHELXTL-PLUS, **2008**, Bruker AXS Inc., Madison, Wisconsin, USA.

(7) Sheldrick, G. M. A short history of SHELX, *Acta Cryst*. **2008**, A64, 112.

(8) Sheldrick, G.M.; Hubshle, C. B.; Dittrich, B. Shelxle: A Qt graphical user interface for SHELXL, *J. Appl. Cryst*. **2011**, *44*, 1281-1284.
